# Comprehensive multi-omics analysis uncovers potential risks of aged sperm on offspring development after short-term storage

**DOI:** 10.1186/s12915-025-02379-5

**Published:** 2025-08-22

**Authors:** Yu Cheng, Songpei Zhang, Sayyed Mohammad Hadi Alavi, Rigolin Nayak, Swapnil Gorakh Waghmare, Nururshopa Eskander Shazada, Deepali Rahi Roy, Zhijun Ma, Otomar Linhart, Zuzana Linhartová

**Affiliations:** 1 Research Institute of Fish Culture and Hydrobiology, South Bohemian Research Center of Aquaculture and Biodiversity of Hydrocenoses, Faculty of Fisheries and Protection of Waters, University of South Bohemia in České Budějovice, Zátiší 728/II, Vodňany, 389 25 Czech Republic; 2 Shandong Provincial Key Laboratory of Animal Biotechnology and Disease Control and Prevention, Shandong Agricultural University, Taian , Shandong, 271018 China; 3 Department of Animal Biology, College of Science, School of Biology, University of Tehran, Enqhelab Avenue, Tehran, 14176-14411 Iran; 4 Neuroscience Research Center, Medical College of Wisconsin, Milwaukee, WI USA; 5 Department of Cell Biology, Neurobiology and Anatomy, Medical College of Wisconsin, Milwaukee, WI USA

**Keywords:** Epigenetics, Fish sperm, Sperm ageing, Offspring development, Epigenetic inheritance

## Abstract

**Background:**

Recent studies have demonstrated that prolonged sperm storage adversely affects offspring through epigenetics, yet its broader effects on other molecular levels such as transcription and proteomics in progeny have been rarely explored.

**Results:**

We employed comprehensive multi-omics approaches to uncover storage-induced epigenetic changes in sperm and their effects on embryonic development and offspring health. Sperm from common carp (*Cyprinus carpio*) was stored in vitro in artificial seminal plasma for 14 days, and the impacts of storage on functional properties of sperm and progeny development were investigated. We combined DNA methylome, transcriptomic and proteomic data to elucidate the potential mechanisms by which sperm storage influences progeny development. Prolonged in vitro storage significantly reduced sperm motility and fertilising ability which coincided with changes in the DNA methylation pattern. Integrated analyses of the offspring DNA methylome, comparative transcriptomics and cardiac performance measurements revealed storage-induced alterations of genes associated with nervous system development, myocardial morphogenesis and cellular responses to stimuli. Proteomic analyses showed that in addition to visual perception and nervous system function, pathways of the immunity system were also enriched. Results provide strong evidence of the epigenetic inheritance of the offspring’s performances when short-term stored sperm was used for fertilisation.

**Conclusions:**

Short-term sperm storage induces heritable molecular and phenotypic changes in offspring, raising concerns over the potential intergenerational consequences of assisted reproductive practices in aquaculture and possibly other vertebrates.

**Supplementary Information:**

The online version contains supplementary material available at 10.1186/s12915-025-02379-5.

## Background

Epigenetic modifications including DNA methylation are highly reactive to environmental stressors (such as temperature and oxidative stress) [1–3]. These external factors can trigger DNA fragmentation, a phenomenon increasingly linked to epigenetic alterations [4, 5]. In recent years, growing evidence has suggested that these epigenetic changes are not restricted to the individual but can be inter- or trans-generationally inherited via gametes upon fertilisation [6–8]. Inheritance of altered epigenetic patterns raises intriguing possibilities about the potential effects on embryonic development, phenotypic changes and the onset of diseases in the offspring [9–12]. Moreover, the sperm epigenome has shown to be sensitive to environmental stress in functional regions, which correlates with phenotypes in the offspring [2, 13–15]. While the generational inheritance of epigenetic modifications has been well documented in mammals and other animal model organisms, the mechanisms of DNA methylation in fish sperm and inheritance of epigenetic alterations to the next generation remain unclear in fishes [16, 17].

Epigenetic mechanisms, including DNA methylation, serve as essential regulators of various biological processes, including transposon silencing and gene expression [18, 19]. However, DNA methylation within gene bodies has been relatively overlooked, despite its potential importance in various biological processes. Genome-wide studies have shown variations in DNA methylation and gene expression in different-aged fishes that may contribute to age-related disease susceptibility [20]. Further, the fate of germ cell DNA methylation during embryogenesis differs across vertebrates. While mammalian embryos undergo global DNA demethylation, the zebrafish (*Danio rerio*) does not, suggesting that sperm methylomes in fishes may be more suited for generational inheritance [21–23].

Interestingly, the most common source of DNA methylome variations in sperm arises during spermatogenesis, particularly when the male is exposed to environmental stressors like nutritional deficiencies [24], pollution [25, 26] or hypoxia [27]. Similarly, direct exposure of sperm to stressors causes epigenetic modifications suggesting that use of cryopreserved or short-term stored sperm in assisted reproductive technologies (ART) results in the inheritance of the phenotype or diseases susceptibility in the progeny [28–31]. In fish farming, the practice of storing sperm for short or long periods is standard [30]; sperm storage allows greater flexibility in timing artificial fertilisation when gametes are unavailable and supports efficient reproductive management such as selective in vitro breeding, disease diagnosis and transportation. However, the risks of passing on epigenetic alterations through sperm storage remain largely unexplored.

A range of paternal factors, including seminal plasma, sperm DNA, proteins and non-coding RNAs (such microRNAs), contribute to shaping embryonic development and reproductive fitness of progeny [32, 33]. DNA methylation, in particular, has been shown to play a key role in ensuring the transcriptional stability of embryos [34]. Recent studies, including work from our laboratory, have delved into understanding storage-induced epigenetic alterations of gametes that may alter developmental pathways in offspring if transmitted at fertilisation [15, 35–38].

Multi-omics technologies have emerged as powerful tools to understand gene expression, epigenetic regulation and even protein profiles across generations [11, 27]. However, comprehensive studies considering sperm storage effects on progeny, especially intergenerational impacts, are still in their infancy. In previous experiments, we observed that extended in vitro storage of sperm for 6 days in common carp (*Cyprinus carpio*) led to alterations in the DNA methylome of the offspring, impacting various biological processes, including cell adhesion and metabolism [15]. At present, advances in fish reproduction suggest manipulation of the storage method makes it possible to store sperm for 14 days while maintaining its functional properties to fertilise oocytes [39–42]. Therefore, it is pertinent to investigate the potential effects of longer storage on the sperm epigenome and the consequences of its alterations on development of embryos and health of progeny. Some key questions that remain to be answered include: (1) Does the extended storage duration of sperm increase the risk of adverse outcomes in offspring? (2) How do storage-induced changes in the sperm epigenome affect offspring phenotypes? (3) Do offspring inherit genes with altered methylation patterns? (4) Do these epimutations impact gene expression and proteins synthesis? In the present study, we aimed to address these questions by investigating the developmental outcomes of offspring derived from sperm stored in vitro for 14 days. Common carp was selected as the model species due to its high economic importance in aquaculture, well-characterised reproductive biology and practical suitability for controlled fertilisation experiments, including sperm manipulation and extended in vitro storage. Using whole-genome bisulphite sequencing (WGBS), RNA sequencing (RNA-Seq) and proteomics tools, we investigated the consequences of prolonged sperm storage on the epigenetics, transcriptomics and the proteomic profile of the resulting embryos with particular emphasis on identifying potential phenotypic changes and developmental defects.

## Results

### Short-term storage negatively affects sperm functional properties and influences offspring development

**Fig. 1:**
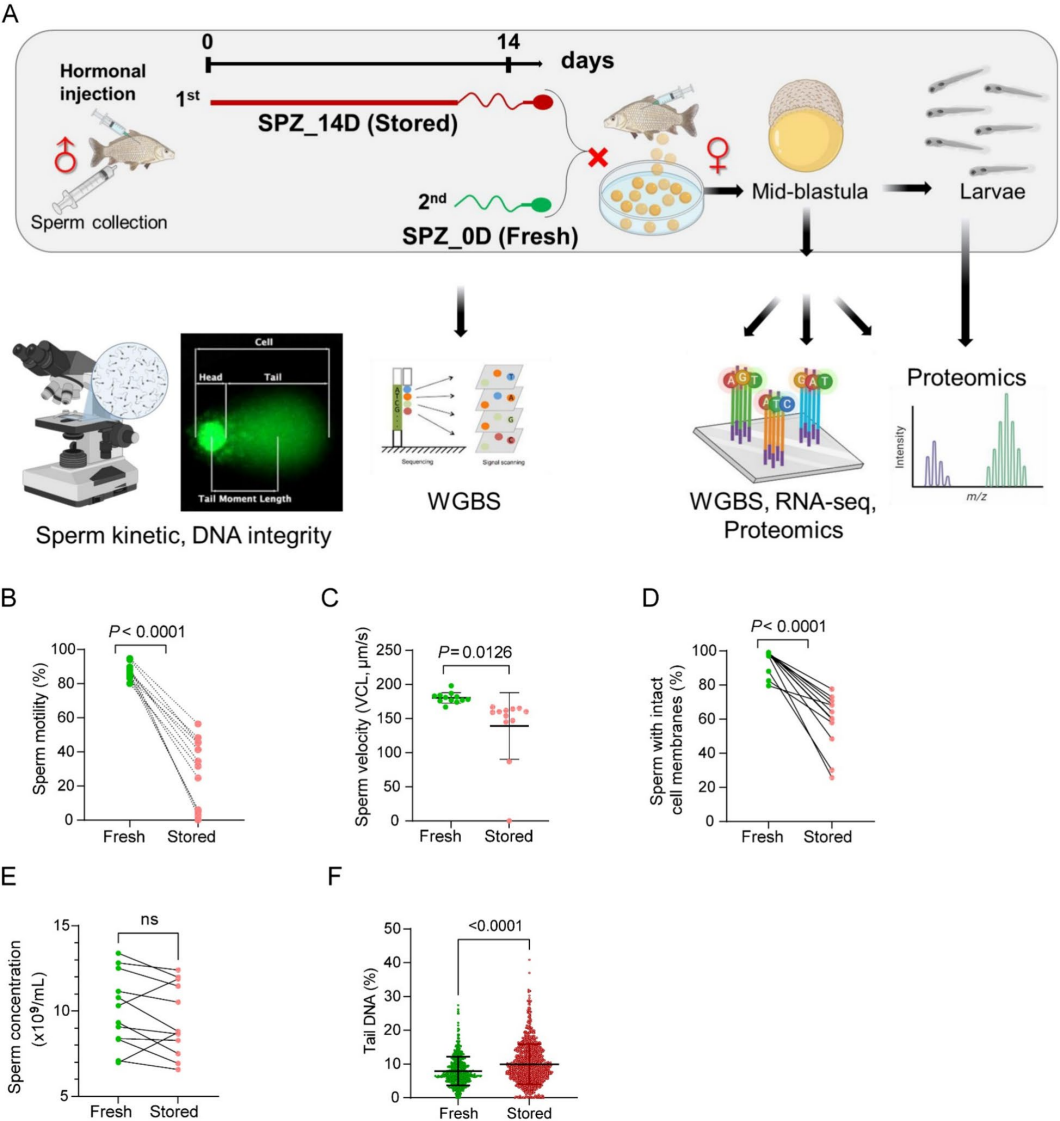
Short-term storage induced reduction of sperm quality and fertilising capacity. **A** The experimental protocol and methods used to study the effects of sperm storage for 14 days on larval development and health perspectives in common carp (*Cyprinus carpio*) using multi-omics approaches. Some elements were created from BioRender. The whole-genome bisulphite sequencing (WGBS), RNA sequencing (RNA-Seq). The effect of short-term storage on sperm motility (**B**), curvilinear velocity (**C**), viability (**D**), sperm concentration (**E**) and DNA fragmentation (**F**). Sperm was stored on ice for 14 days, and motility analysis was performed using a CASA. Results are expressed as means ± S.D., and actual *P* values are shown (*n* = 12)

The effects of storage on functional properties of sperm were compared between fresh sperm and sperm stored for 14 days in vitro (Fig. 1A). Sperm motility and velocity parameters (such as VCL and VAP) were significantly lower in 14-day-stored sperm than fresh sperm (Fig. 1B, C and Additional file 1: Fig. S1). However, some sperm motility and velocity parameters (VSL, LIN, WOB, PROG and BCF) remained unaffected (Additional file 1: Fig. S1). VCL and VAP, reflecting sperm’s velocity and curvilinear movement, may decrease during storage due to lower energy and membrane integrity. In contrast, VSL, LIN and PROG, which reflect directionality and progressive movement, may remain stable as they are less dependent on high energy levels and more resilient to short-term storage. It suggests that storage primarily affects motility intensity while leaving directional swimming and progressive movement largely unaffected.

The storage-induced reduction of sperm motility and velocity was accompanied by decrease in membrane integrity which is an indicator for sperm health and fertilisation ability (Fig. 1D). Additionally, a reduction in sperm concentration was observed across most males (Fig. 1E), further highlighting the negative effects of storage. Importantly, storage of sperm increased DNA fragmentation (Fig. 1F). Both decrease in membrane integrity and increase in DNA fragmentation are signs of damages to the sperm at cellular and molecular levels. The damage to genetic material raises concerns about potential epigenetic variations and transmission of these defects to the next generation.

**Fig. 2:**
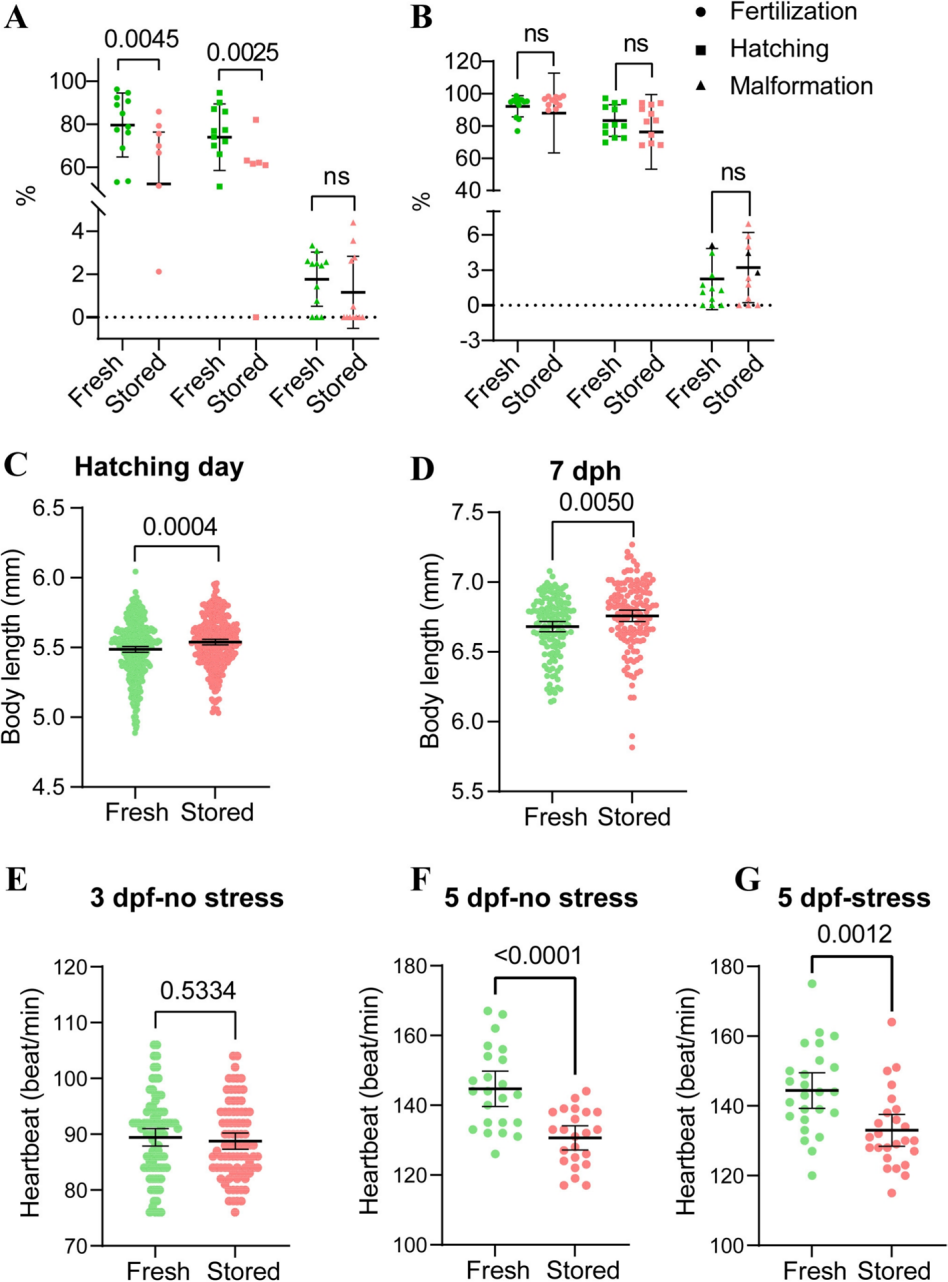
Storage effects on sperm fertilising ability and embryonic development of larvae in common carp (*Cyprinus carpio*). Sperm was stored on ice for 14 days, and fertilisation was performed at 600,000 sperm/egg (**A**) and 3,000,000 sperm/egg (**B**). The body length (mm) of offspring was measured at hatching day (**C**) and 7 days post-hatching (dph) (**D**). For cardiac performance, the heartbeat rate was measured at 3 and 5 days post-fertilisation (dpf) under rest conditions (**E** and **F**) or under a 60-s acute stress exposure (**G**). Results are expressed as means ± S.D., and actual *P* values are expressed (F0 male: *n* = 12 for **A** and **B**, *n* = 4 for **C**–**G**)

These molecular and physical changes of aged sperm had direct impacts on fertilisation outcomes. Storage of sperm resulted in lower fertilisation and hatching rates when fertilisation was performed at 600,000 sperm cells per egg (Fig. 2A). Increasing the sperm:egg ratio to 3,000,000 masked negative effects of storage on sperm fertilising capacity (Fig. 2B). Interestingly, no significant deformities were observed in the offspring at the hatching stage (Fig. 2A, B), suggesting that the initial effects of sperm storage do not immediately manifest themselves in severe physical abnormalities.

Nevertheless, the offspring from the aged sperm group exhibited differences from fresh sperm during development. Offspring from the former were significantly longer in body length in the early stages, such as on hatching day and 7 dph (Fig. 2C, D), indicating that aged sperm may enhance early growth rates in offspring. It may be attributed to storage-induced epigenetic modifications or selection of more resilient sperm that fertilise the eggs. These changes could influence early gene expression, promoting faster growth and development in the embryo. Along with changes in body size, cardiac performance (heartbeat) was reduced in the offspring from aged sperm, irrespective of external stimuli at 5 dpf, while similar at 3 dpf (Fig. 2E–G). Taken together, these results suggest that while aged sperm probably promotes intrinsic growth at an early stage, it comes at a cost, however, as evidenced by potential dysregulation in cardiac function.

### Short-term sperm storage alters DNA methylation in sperm and resulting offspring

**Fig. 3:**
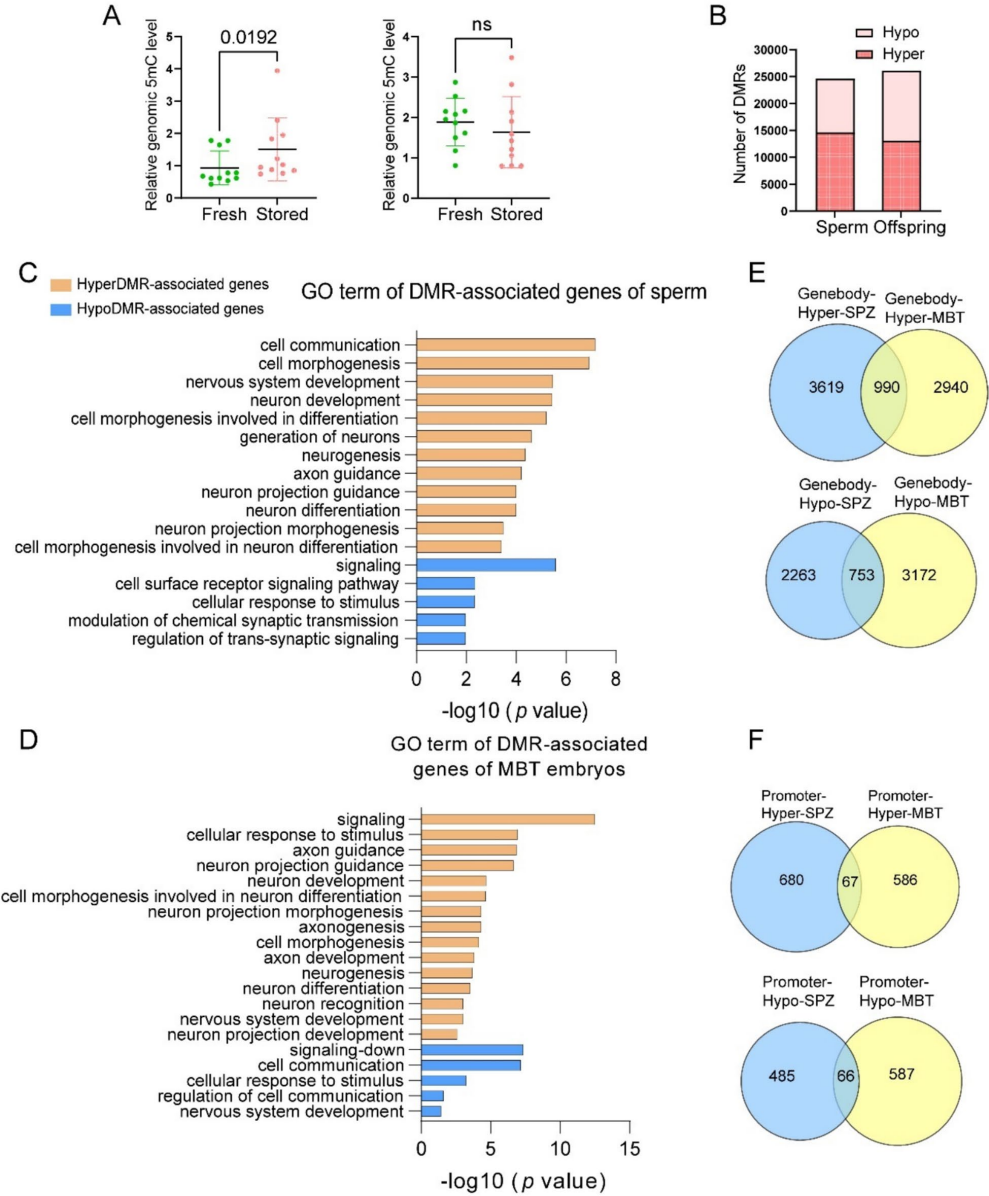
**A** Effects of storage on global DNA methylation (5mC%) of sperm (left) and embryos at the mid-blastula stage (right) (*n* = 12). **B** Number of differentially methylated regions (DMRs) (Hypo, hypomethylated; Hyper, hypermethylated) and their associated genes in sperm and resulting embryos, showing numbers of DMRs in sperm and offspring differed between fresh sperm and 14-day-stored sperm. Effects of storage on the DMR-associated genes in sperm (**C**) and resulting embryos at mid-blastula stage (**D**) using GO term analysis. Venn diagrams indicate the number of shared and unique hyper- and hypo-DMRs-associated genes between sperm and embryos of the gene body (**E**) and promoter (**F**)

We investigated the effects of short-term storage on DNA methylation in sperm and investigated whether the changes are transmitted to the next generation. We used ELISA assays and WGBS to compare DNA methylome between fresh and 14-day-aged sperm and their offspring (Fig. 3A). Storage resulted in an increase in the 5mdC level in sperm (*P* = 0.0192), while 5mdC level did not differ between the derived embryos. While global DNA methylation changes provided a limited understanding of the molecular mechanisms, WGBS analysis revealed high-quality methylation data in a single base with a bisulphite conversion rate exceeding 99.45% and a mapping rate of over 76% to the common carp reference genome (GCF_018340385.1, Additional file 2). We found that genomic DNA in both sperm and F1 embryos was globally hypermethylated, with CpG methylation levels averaging *c.* 93%. Additionally, CHG and CHH methylation levels were *c.* 1.4% and 4%. The embryos at the mid-blastula stage exhibited methylation levels similar to those observed in sperm (Additional file 2; Additional file 1: Fig. S2A–H), suggesting that DNA methylation patterns in sperm may be transmitted to early embryos.

Bisulphite sequencing revealed extensive differential methylation in both sperm and their offspring. We identified 24,583 DMRs in aged sperm compared to fresh sperm with 14,600 hypermethylated and 9983 hypomethylated DMRs (Fig. 3B). In the embryos, 26,109 DMRs were observed with nearly equal numbers of hypermethylated (13,030) and hypomethylated (13,079) regions (Fig. 3B; Additional file 2; Additional file 1: Fig. S2I, J). These findings indicate storage-induced extensive methylation changes in both sperm and embryos.

Further analysis of the DMR-associated genes revealed that 7625 genes in the aged sperm were linked to these regions, including 4609 hypermethylated and 3016 hypomethylated genes. In the offspring, 7855 DMR-associated genes were identified with 3930 hypermethylated and 3925 hypomethylated (Additional file 2). This significant overlap suggests that aged sperm may carry forward and transfer epigenetic markers to influence the development of the next generation.

To investigate the functional implications of these methylation changes, we performed GO and KEGG pathway analyses in both gene body and promoter regions. In the gene body region of stored sperm, hypermethylated DMR-associated genes were enriched in processes such as nervous system development and cell communication and morphogenesis (Additional file 3; Fig. 3C), while hypomethylated genes were linked to synaptic transmission and signalling pathways (Additional file 3; Fig. 3C). Similarly, in the mid-blastula embryos, hypermethylated genes were involved in cellular responses to stimuli, cell morphogenesis and axonogenesis, while hypomethylated genes were enriched in processes related to signalling pathways, cell communication and nervous system development (Additional file 4; Fig. 3D). These shared enrichment patterns between sperm and offspring highlight the potential role of sperm DNA methylation in embryonic development, with many DMR-associated genes in sperm being resistant to reprogramming in the early embryo.

We also analysed the promoter regions of the genome defined as regions within 1 kb of the transcription start site (TSS). Only one GO term, the L-methionine salvage from S-adenosylmethionine, was enriched in hypermethylated DMR-associated genes in sperm (Additional file 3). Additionally, 990 hypermethylated and 753 hypomethylated gene body DMR-associated genes were shared between aged sperm and their embryos (Fig. 3E; Additional file 2), indicating common methylation patterns that are passed on to the next generation. These common genes were further analysed alongside transcriptome data from offspring to assess their functional significance (Table 1). In promoter regions, 67 hypermethylated and 66 hypomethylated DMR-associated genes were identified in both sperm and offspring groups (Fig. 3F; Additional file 2). Taken together, results so far show short-term storage-induced epigenetic changes in sperm, specifically in DNA methylation, which are transmitted to offspring. These alterations play key roles in developmental processes which affect development of the embryos.

**Table 1:** List of common genes in the differentially methylated regions (DMRs)-associated from sperm, embryos and RNA expression in embryos

Gene	Methylation in sperm	Methylation in embryos	mRNA in embryos
*itgb1*		Up	Up
*notch1*		Down	Down
*huwe1*		Down	Down
*baz2a*	Down (gene body hypo)	Down	Down
*cops6*	Up (promoter hyper)	Down	Up
*birc6*		Down	Down
*nbeal1*		Up	Up
*tjp2a*		Down	Down
*flnb*		Down	Down
*chd7*	Down (gene body hypo)	Up	Down

### Transcriptomic analysis reveals functional impacts of sperm storage on the embryo’s development

**Fig. 4:**
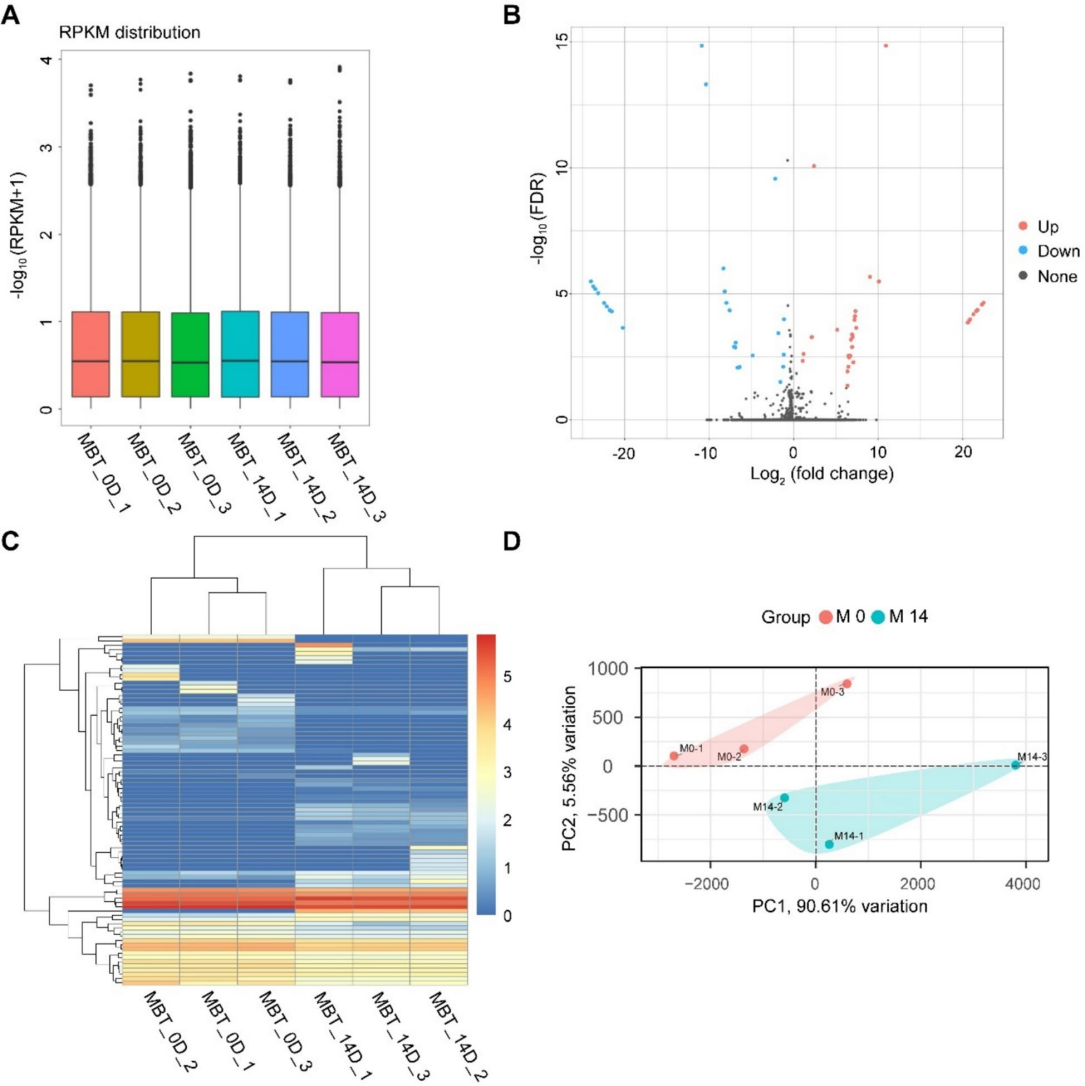
Effects of short-term sperm storage on differentially expressed genes (DEGs), common genes in differentially methylated regions and RNA expression in the resulting embryos. **A** Reads Per Kilobase per Million mapped reads (RPKM) distribution in each sample. **B** Upregulated and downregulated DEGs in the embryos. **C** Hierarchical clustering heat map for the relative levels of gene expression. **D** Principal component analysis of the transcriptomes of F1 offspring

We performed transcriptomic analysis to investigate the effects of sperm storage on the functional consequences of the altered methylation patterns in the offspring (Fig. 4A). This analysis provides valuable information to understand the association between the methylation pattern and gene expression. We obtained 44.33 M and 44.27 M quality trimmed reads, which translates to 6.65 Gb and 6.64 Gb of total clean bases from both fresh and 14-day-aged sperm. A total of 83 DEGs were identified in the embryos resulting from 14-day-aged sperm, of which 39 and 44 genes were respectively upregulated and downregulated in embryos at the mid-blastula stage (Fig. 4B, C; Additional file 5). This suggests translation of the storage-induced epigenetic changes in the embryos during early development. Principal component analysis (PCA), especially in the second principal component (PC2), revealed a distinction between the embryos resulting from 14-day-aged sperm and those derived from fresh sperm (Fig. 4D). This separation indicates that the transcriptomes of the embryos are significantly altered by the storage-induced changes in sperm.

GO term and KEGG pathway enrichment analyses of DEGs identified in embryos derived from 14-day-aged sperm revealed that these genes were involved in critical developmental processes including myocardium morphogenesis and nerve development (Additional file 1: Fig. S3). The circadian rhythm pathways, which play critical roles in regulating both cardiac and neural functions, were also impacted. These findings suggest that the transcriptomic changes in the embryos are not random but linked to specific systems, particularly the heart and nervous system, that are crucial for proper development. This is consistent with the observed cardiac performance deficits in offspring, reinforcing the connection between altered gene expression and functional impairments. Taken together, the results highlight that the storage-induced transcriptomic changes in sperm may contribute to significant functional impairments in early development, potentially leading to long-term physiological consequences for the offspring.

### Sperm storage disrupts protein networks and affects early offspring development

**Fig. 5:**
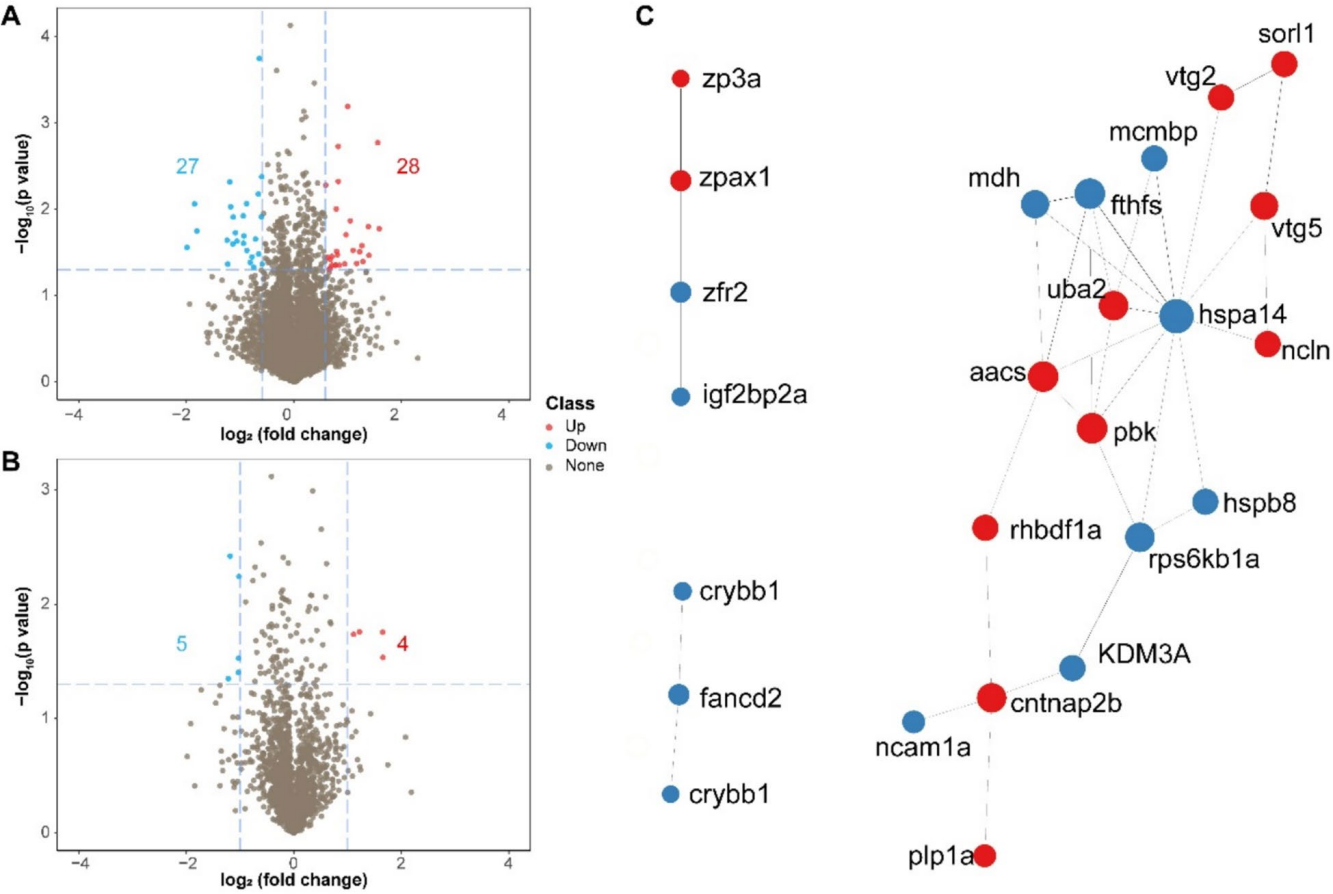
Sperm storage induces differentially expressed proteins (DEPs) in the embryos. Volcano plots illustrate the distribution of all proteins identified with the LC–MS/MS approach in the larvae (**A**) and embryos at the mid-blastula stage (**B**). Significantly up- and downregulated proteins (|fold change|≥ 1.3, *x*-axis; FDR adjusted *P* value ≤ 0.05, *y*-axis) are highlighted in green and red, respectively. Statistically up- and downregulated proteins with non-significant biological changes (|fold change|< 1.3) are shown in light green and orange, respectively, and proteins with non-significant differences between the free and encapsulated yeast are shown in grey. **C** Protein–protein interaction (PPI) networks among differentially abundant proteins of the larvae derived from fresh and stored sperm. PPI analysis of DEPs was done by searching STRING PPI database, and the top 100 interactions with confidence were used to construct the interaction map. Red nodes represent proteins with upregulation; blue nodes represent proteins with downregulation. The size of the circle indicates the density of relationship

To explore the proteomic alterations induced by sperm storage and how these changes relate to the phenotypic differences observed in F1 offspring (embryos at hatching and mid-blastula stage), we performed an in-depth proteomic analysis using LC–MS/MS in the DIA mode for relative quantification. A total of 6068 proteins were identified in the hatched embryos, and a significant difference in protein expression was observed between the hatched embryos produced from fresh and 14-day-aged sperm (Fig. 5A). These discrepancies in protein expression suggest that sperm storage induces molecular changes passed on to their progeny by finally acting on protein expression.

To better understand which expression pathway was affected by storage, we classified the differentially expressed proteins according to their functions and the pathways in which they are involved. The analysis revealed that a large proportion (over 20%) of the affected proteins were membrane components primarily involved in antigen processing and presentation pathways. These pathways play crucial roles in regulating the immune response, including antigen presentation and immune system regulation, which can have implications for disease susceptibility (Additional file 6). Protein lists were selected based on a *P* < 0.05 cutoff for pathway enrichment analyses (Partek Genomics Suite 6.6; Partek, Inc., St Louis, MO, USA). Interestingly, several enriched proteins were linked to visual perception and nervous system function (Additional file 6), which regulate fish behavioural adaptation and sensory regulation [43]. This could help explain the reduction in heartbeat observed in larvae under external stress, as these sensory systems may be impaired in the larvae derived from 14-day-aged sperm. Processes like neurogenesis and neural plasticity, both of which are vital for sensory and movement control, could be also disrupted by sperm storage, potentially affecting neurological function and development [44].

In addition to the larval proteome, we also analysed the proteomic differences in F1 embryos at the mid-blastula transition (MBT) stage. This was done to maintain consistency with the other omics analyses. A total of 2674 proteins were identified at the MBT stage, with only four upregulated and five downregulated proteins (Fig. 5B). These proteins were primarily enriched in protein-DNA complex, chromosome structure and pathways related to the processes of replication, repair and transcription (Additional file 6), aligning with the observed transcriptional changes.

To further probe the impact of sperm storage on early development, we constructed a protein–protein interaction (PPI) network to visualise how the differentially expressed proteins interact functionally (Fig. 5C). The PPI network revealed several key interaction hubs, with hspa14 at the centre, interacting with multiple proteins, including ubiquitin-like modifier-activating enzyme 2 (*UBA2*), nicalin (*NCLN*) and vitellogenin 2 (*VTG2*). The upregulation of hspa14 and its central role in the network suggest that pathways involved in heat shock response and protein folding are significantly altered in embryos derived from 14-day-aged sperm. This could indicate increased cellular stress during early development in the preserved sperm group.

The PPI network highlighted the upregulation of vitellogenins, particularly *VTG2* and *VTG5*, which regulate yolk formation to provide early embryonic nutrition. The significant upregulation of these proteins in the embryos from 14-day-aged sperm suggests alterations in the nutritional support mechanisms. Notably, proteins such as *ZFR2* and *CNTNAP2B* involved in RNA processing and neural development were downregulated, indicating potential disruptions in pathways critical for neural development. Additionally, the epigenetic regulator KDM3A was found to interact with proteins related to gene expression, pointing to possible epigenetic changes linked to sperm storage.

### Omics integration reveals storage-induced epigenetic inheritance mechanisms

Finally, we integrated the methylome and transcriptome data to identify common genes affected by both sperm and embryo DNA methylation, then gene expression changes in F1 offspring. We identified 10 common genes between the DMGs and DEGs (Additional file 1: Fig. S4, Table 1, Additional file 7). These shared genes, such as *itgb1* and *notch1*, suggest that the epigenetic modifications observed in aged sperm are potentially passed on to the offspring, influencing their early development. For example, *itgb1* exhibited increased methylation and expression, while *notch1*, *huwe1* and *baz2a* were downregulated in both methylation and mRNA levels in embryos. Notably, *cops6* displayed increased sperm and embryo methylation and mRNA expression in embryos, while *tjp2a* and *flnb* showed decreased methylation and expression, whereas *chd7* was hypomethylated in sperm while hypermethylated in embryos.

The overlap between methylation and gene expression patterns suggests a clear mechanism of epigenetic inheritance. The altered methylation profiles in 14-day-aged sperm were inherited and affected the early gene expression in embryos, particularly in genes related to neural and cardiac development. This supports the idea that sperm storage not only influences immediate reproductive outcomes but also has lasting effects on the next generation through inherited epigenetic changes.

## Discussion

### Sperm storage-induced acceleration in early development coincides with impairment of growth

Our study demonstrates that sperm storage impacts sperm quality with subsequent effects on embryonic development, while does not cause visible embryos and larvae malformations. Results show the adverse effects of storage are mediated through epigenetic alterations and changes in protein expression of offspring. We observed a reduction of cardiac performance in larvae derived from the aged sperm, particularly when larvae were subjected to external stress, suggesting potential impairments of the cardiovascular and nervous systems. However, embryos resulting from aged sperm showed accelerated early development that was about 1.0% faster than their counterparts produced from fresh sperm. These findings suggest that aged sperm might promote endogenous growth up to hatching, and raise the question of whether this acceleration has long-term developmental trade-offs that emerge later in the larval stage or adulthood. It should be noted that these data were only found in the offspring of one female, so they can be considered speculative, and need to be investigated on a larger sample of parents.

Previous research has extensively documented that sperm storage leads to significant detriments in sperm motility, concentration and DNA integrity across several species, including humans, primates, boars, rams, mice and fishes [15, 37, 41, 45–52]. These studies have established that sperm exposed to storage display increased DNA fragmentation along with generation of reactive oxygen species. These lead to broad epigenetic and metabolic alterations and a subsequent decline in fertilisation rates. Our findings are consistent with these reports showing storage effects on sperm quality. The present study extends these findings by focusing on the intergenerational effects of sperm storage, revealing that epigenetic changes in aged sperm are passed down to offspring, leading to observable developmental changes at both phenotypic and molecular levels.

### Use of aged sperm in fertilisation alters offspring growth at endogenous early developmental stages

The observed growth advantage in embryos produced from aged sperm is particularly interesting, especially as it was only present during the early stages of development. Early growth acceleration has been previously observed in other species. For example, in Eurasian perch (*Perca fluviatilis*), larvae produced from cryopreserved sperm exhibited faster growth rates during early development, which some studies attribute to enhanced eyesight that facilitates more efficient foraging [53]. We found that this endogenous growth advantage may be linked to differences in yolk absorption and metabolic efficiency, as suggested by GO terms related to fat digestion, absorption and glycerolipid metabolism in the transcriptomic analysis. These metabolic pathways play functional roles in the early developmental stages, especially during the endogenous nutrition phase prior to mouth opening [54]. Further studies are needed to determine whether the growth differences persist after the larvae initiate external feeding and even whether these variations in growth potential extend over several years under consistent feeding and environmental conditions. The potential changes may be attributed to alterations in nutrient metabolism or energy allocation [55]. In addition, the differential regulation of the blue light signalling pathway observed in this study, which is known to influence eye development, may play a potential role in balancing metabolism following the switch to exogenous feeding. Taken together, these findings highlight the complexity of developmental responses to sperm storage, which may vary across species and developmental stages. For example, in other fish species, such as brown trout (*Salmo trutta*), embryos produced from aged sperm exhibited slower growth after hatching [56].

### Sperm storage induces epigenetic remodelling with lasting developmental effects

Another critical finding in our study was the extensive epigenetic remodelling observed in aged sperm. Previous studies have demonstrated that sperm stored for a shorter period of time (e.g. 6 days) exhibit minimal changes in global DNA methylation [37]. However, in our study, sperm stored for 14 days exhibited a significant increase in DNA hypermethylation. This suggests that the increase in the duration of storage triggers massive epigenetic changes, particularly in DNA methylation patterns. These changes could have important implications for ART, not only in fishes but also in other vertebrates, including humans and mice. Epigenetic changes such as DNA methylation are known to be critical regulators of gene expression, and alterations in these patterns during sperm storage could influence a wide range of developmental processes in offspring [17].

When taking into account DNA methylation in a single base, our analysis of DMGs revealed that both aged sperm and their offspring shared enrichment in GO terms related to cell morphogenesis, neuron development and cellular responses to stimuli. This overlap suggests that epigenetic changes in sperm may be transmitted to the next generation and persist through early stages of embryonic development. Previous studies in zebrafish have demonstrated that sperm methylation patterns are largely retained in embryos during the mid-blastula stage [22, 23], supporting the idea that paternal epigenetic information escape reprogramming during embryogenesis and pass down to offspring. Our findings provide novel insights, demonstrating that embryos developed from aged sperm in common carp exhibit similar methylation retention, which may influence the developmental trajectory of the next progeny.

The epigenetic changes observed in the common DMGs in sperm and embryos such as *cops6*, *baz2a* and *chd7* are of particular interest due to their known roles in development. *Cops6* plays a critical role in early embryonic development including dorsoventral patterning and cardiac development [57, 58]. The differential methylation of *cops6* in aged sperm and its offspring suggests that this gene may contribute to the observed cardiac impairments in the offspring. Interestingly, bromodomain adjacent to zinc finger (baz) protein domain genes such as *baz2a* are acetyl-lysine specific epigenetic reader domains in the epigenome [59]. Moreover, single nucleotide polymorphisms (SNPs) within the *baz2b* gene locus have been linked to sudden cardiac death [60]. Similarly, *chd7*, which is essential for cardiovascular and neural development [61], exhibited hypermethylation in offspring, correlating with reduced transcriptional activity and potentially contributing to the reduced cardiac performance observed in the offspring. The observed epigenetic modifications in these genes support the hypothesis that sperm storage-induced methylation changes are passed down to offspring, influencing key developmental processes related to heart and neural function [59–61].

### Multi-omics integration reveals the broad impacts of sperm storage on offspring development

Transcriptomic analysis provided further insights into the functional impacts of these epigenetic changes on gene expression. Genes such as *itgb1*, *notch1* and *huwe1* exhibited significant methylation changes, which correlated with altered transcriptional expression in offspring. These genes are known to be involved in neural development and early differentiation, suggesting that the observed epigenetic alterations in aged sperm may disrupt these critical developmental pathways. For instance, *notch1* regulates neural differentiation and has been shown to influence cardiogenesis during embryonic development [62, 63]. The downregulation of *notch1* in offspring from aged sperm indicates that these epigenetic changes may contribute to developmental delays or functional impairments of the nervous and cardiovascular systems.

In addition to the epigenetic and transcriptomic changes, our proteomic analysis revealed significant alterations in protein networks related to immune responses, visual perception and neural development. Key proteins such as *HSPA14* and vitellogenin were upregulated in offspring from aged sperm, indicating an increased cellular stress response and alterations in yolk formation during early development. *HSPA14* functions in protein folding and the heat shock response. Our results suggest that sperm storage may induce stress-related protein changes that affect offspring resilience to environmental stressors. Meanwhile, the upregulation of vitellogenin highlights potential changes in nutritional support mechanisms during early embryonic development. Additionally, downregulation of *ZFR2* and *CNTNAP2b* in offsprings that involve RNA processing and neural development suggests potential disruptions of neural pathways [64, 65].

Further, the PPI network reveals that key processes related to protein folding, nutritional support and neurodevelopmental pathways were significantly affected by sperm storage. These proteomic changes, combined with earlier epigenetic and transcriptomic findings, highlight how sperm storage introduces molecular disruptions that manifest as physiological and developmental impairments in offspring. This emphasises the long-term consequences of sperm storage on early development, demonstrating that the molecular legacy of aged sperm can shape the fate of future generations.

The integration of methylome, transcriptome and proteome data in this study provides a comprehensive view of sperm storage impacts on offspring development at multiple molecular levels. The observed molecular alterations were consistent with the phenotypic changes in offspring as seen in cardiac performance and early growth patterns. These findings emphasise the importance of considering the long-term effects of sperm storage on reproductive outcomes, not just in terms of immediate fertilisation success, but also in relation to offspring health and development [27, 66–68]. Looking ahead, further research is needed to validate these findings, including functional assays to confirm the roles of key differential genes and proteins in embryos. Understanding these mechanisms in greater detail will provide deeper insights into how sperm preservation practices affect offspring development and fitness, guiding future efforts to mitigate the potential risks of artificial reproductive technologies.

## Conclusions

This study offers the first evidence that vertebrates can modify their epigenomes in response to stress during sperm storage, passing these epigenetic changes through the male germline to affect offspring. Our findings reveal that aged sperm induces a series of DMRs in parental sperm, which are subsequently transmitted to the offspring. The storage-induced changes in sperm disrupt the methylome, transcriptome and proteome in the offspring. In this study, we reported storage-induced effects on genes and proteins associated with the nervous and cardiac systems. The transmission of altered epigenetic information led to modifications in key genes, such as *itgb1*, *notch1*, *huwe1*, *nbeal1* and *chd7*, contributing to functional impairments in these vital systems.

The observed defects in neural and cardiac function in offspring suggest that sperm storage has far-reaching developmental consequences, highlighting potential risks of artificial reproductive techniques to the sustainability of fish populations. Our data underscore the importance of assessing the full impact of sperm storage, not just on immediate fertilisation success, but on the long-term health and viability of offspring. The observed epigenetic, transcriptional and proteomic changes may contribute to critical defects that potentially threaten overall population health.

## Methods

### Ethics approval

All procedures employed in this study for animal handling and sampling were conducted in accordance with the relevant legal and ethical standards. The breeding and supply of animals was authorised by the Ministry of Agriculture of the Czech Republic under reference number 64155/2020-MZE-18134. The use of animals for research was authorised under reference number 68763/2020-MZE-18134. The authors of this study, O.L. (CZ 02815), hold Certificates of Professional Competence in accordance with Sect. 15d(3) of Act No. 246/1992 Coll. on the Protection of Animals against Cruelty, which qualifies them to perform animal experiments.

### Broodstock and gamete collection

A total of 12 five-year-old mature male common carp, with body weights ranging from 1.5 to 3 kg, were selected from a recirculation aquaculture system. The fish were regularly fed, in good health and produced high-quality sperm. A single intramuscular injection of carp pituitary dissolved in physiological saline was performed at 2 mg/kg body weight to stimulate sperm maturation [69]. One day post-hormonal treatment, each male was stripped for sperm using a syringe. The sperm was placed on crushed ice (0–2 °C) under aerobic conditions and transferred to our Reproductive Physiology Lab for quality assessment. Sperm samples with motility > 80% were selected for the experiment. The sperm motility was evaluated using the CASA system, following the protocol described in Cheng et al. (2023, 2024). To conduct the fertilisation test, female specimens (7-year-old, ~ 8 kg) in optimal physical condition were selected in May and stocked separately in the hatchery in 4-m^3^ tanks with a water flow rate of 0.2 L/s, temperature of 18–22 °C and 6–7 mg/L O_2_. Finally, one female was used to stimulate final maturation of oocytes with double administration of CP: 0.5 mg/kg body weight at the primary injection and 2.7 mg/kg body weight at the second injection (the time between the primary and second CP administration was 12 h).

### Sperm storagein vitro

The experiment was designed to obtain fresh sperm and in vitro stored sperm from the same male to investigate the effects of storage on sperm functional properties and its consequences on epigenetics, transcriptomics and proteomics landscapes of the resulting embryos. To standardise the protocol and minimise differences from the mother’s side, fertilisation was performed using oocytes of one female. The procedure involved multiple hormone injections and male stripping, which were carried out with great care to avoid harming the fish. Collected sperm from each specimen was diluted with an extender (110 mM NaCl, 40 mM KCl, 2 mM CaCl_2_, 1 mM MgSO_4_, 20 mM Tris, pH 7.5 and 310 mOsmol/kg) [39] at a ratio 1:1 (*v*:*v*). Fresh sperm and stored sperm on ice for 14 days were assessed for motility kinetics and fertilising ability.

### Evaluation of sperm phenotypes, concentration and DNA fragmentation

Sperm samples were evaluated for phenotypic traits including motility, velocity, membrane integrity and DNA fragmentation [15, 37]. To significantly enhance sperm quality, both fresh and stored sperm were incubated at room temperature (21 °C) for 20 min prior to the analysis of sperm motility kinetics [42]. Distilled water containing 0.25% Pluronic F-127 was used as the activation solution. The motility kinetics of sperm was recorded at 15 s post-activation utilising the CASA plugin for ImageJ [70]. Percentage of motile sperm (MOT), straight-line velocity (VSL), curvilinear velocity (VCL), average path velocity (VAP), linearity (LIN), wobble (WOB), progression (PROG) and beat cross frequency (BCF) were analysed for each sperm sample.

The integrity of the sperm membrane was determined using the Live/Dead Sperm Viability kit (L-7011, Molecular Probes) according to Flajšhans et al. [71] and Li et al. [72] with some modifications. In brief, sperm was initially diluted with an immobilising solution [39] to reach a suspension with 5 × 10^6^ spermatozoa/mL. Next, 0.2 μL SYBR 14 dye and 2.5 μL propidium iodide were added into a 500 µL sperm suspension. Ten microlitres of sperm suspension was incubated on a glass slide with a cover for 10 min in the dark under a fluorescence microscope (OLYMPUS BX50, Japan). The images were analysed for the number of live (green-labelled cells with intact membranes) and dead (red-labelled cells with damaged membranes) sperm using the ImageJ software. The proportion of live spermatozoa was calculated from 15 fluorescent images per sample.

The concentration of sperm was quantified using a Bürker counting chamber. The samples were diluted in 0.9% (w/v) NaCl (physiological solution, PS) by a factor of 1:100 (sperm: PS), and a secondary dilution was performed by 1:50 (sperm: PS). The spermatozoa were counted in 12 squares under the microscope with optical negative phase-contrast and × 10 magnification, with each measurement performed in duplicate.

The examination of sperm DNA fragmentation was conducted in accordance with previously reported methodology [15]. Briefly, the DNA integrity of sperm was assessed with a modified alkaline comet assay. Microscope slides were pre-coated with 1% normal-melting-point agarose in TBE buffer a day prior to analysis. Sperm (0.6 µL) was diluted in 5 mL of phosphate buffered saline (PBS; P-4417, Sigma-Aldrich; prepared at a concentration of 1 × and pH 7.4), then mixed with agarose and solidified on slides at 4 °C. The slides were immersed in lysis buffer overnight, washed and subjected to electrophoresis in the TBE buffer for 15 min. The DNA was stained with SYBR Green, and images were captured using fluorescence microscopy, then analysed with Comet Assay Software Project (CASP) software Project Lab (CASPLab 1.2.3 beta 2).

### Artificial fertilisation, embryo incubation and growth potential

For fertilisation, the concentration of sperm was quantified according to the above method. Individual fresh and stored sperm samples were used separately to assess the effects of sperm storage on fertilisation success. Each sample was tested independently to ensure accurate comparison between fresh and stored sperm quality. To guarantee the highest fertilisation, 1 g of eggs (*c.* 800 eggs) was fertilised at a ratio of 3,000,000 sperm cells per egg [69] with 5 mL activation solution (45 mM NaCl, 5 mM KCl, 30 mM Tris, pH 8.0, 160 mOsmol/kg) [73]. To ascertain the fertilisation potential of stored sperm, a reduced ratio of sperm cells to eggs was employed. In this regard, 0.1 g of eggs (*c.* 80 eggs) were fertilised at ratio of 600,000 sperm cells per egg with 2 mL of activation solution. The fertilisation test was performed in a beaker, and for each male, eggs fertilised with fresh and stored sperm were incubated in four Petri dishes per treatment at 23 °C in hatchery water (pH 7.9, 4 mOsm/kg) [74].

Ratios of fertilisation, hatching and malformation: The embryos in Petri dishes were photographed by a SZ608T stereomicroscope at the eye-stage for fertilisation rate assessment. Hatched embryos were manually counted. Fertilisation rate (fertilised eggs/total eggs * 100), hatching rate (hatched embryos/total eggs * 100) and malformation rate of embryos (abnormal hatched embryos/total hatched embryos * 100) were calculated for fresh and stored sperm groups. The malformed hatched embryos were in unusual body proportions, e.g. peritoneal or heart oedema, irregular body axis and head malformities.

After hatching, Petri dishes were removed and hatched embryos (150–250 individuals) were transferred to a plastic box containing 2 L of hatchery water for continued incubation at 23 °C. Throughout the embryo incubation period, two-thirds of the hatchery water was refreshed daily to maintain optimal water quality and stable environmental conditions. Following fertilisation, embryos at the mid-blastula stages (5.5 h post-fertilisation (hpf)) and hatched embryos on the day of hatching were collected for molecular study.

Cardiac performance: We measured heartbeat to characterise the cardiac performance. The heart rates (beats per minute; bpm) of the embryo generated from fresh and stored sperm were counted by viewing under the SZ608T stereo microscope at rest. Developmental stages were chosen 3 and 5 dpf, meaning before and after hatching. Then, we conducted the stress exposure experiment, which consisted of placing 30 embryos at 5 dph in a beaker with 80 mL of hatchery water, at 23 °C and swirling them with a plastic pipette once every second for 60 s. The embryos were maintained at 23 °C in a beaker over the course of exposure to the stressor. After swirling, individual embryos were transferred to 60-mm Petri dishes (Fisher Scientific, Ottawa, ON) in hatchery water for heartbeat assessment. The heart rates were counted at 5 s post-stressors.

Growth traits of embryos and larvae: On the day of hatching (5 dpf), embryos were transferred to a plastic box (28 cm × 21.5 cm × 7.5 cm) [74] filled with 2 L of hatchery water at 23 °C. Embryos (*n* = 30/group), after anaesthesia (0.05% tricaine methanesulfonate, MS-222) in Petri dishes, were photographed in a custom-made photo box with a dissecting microscope (NSZ608T) for embryo measurements. The larvae were photographed again at 7 dph. The standard length of each larva was measured in ImageJ [75].

### DNA extraction and preparation

Genomic DNA was extracted from 2 µL sperm samples obtained from three males using a DNA Mini Kit (Invitrogen, USA), or extracted from 50–60 mid-blastula embryos and 10–20 embryos at hatching using SDS-based methods as previously reported [37, 76]. The genomic DNA degradation and contamination were evaluated by 1.5% agarose gel electrophoresis to check the DNA quality for library construction. The concentration of DNA was quantified utilising the Nanophotometer Pearl (Implen, Munich, Germany).

### Quantification of genome‐wide DNA methylation

The global DNA methylation (5-mC level) in sperm and resulting embryos was quantified by an enzyme‐linked immunosorbent assay (EpiGentek MethylFlash P-1030) according to the manufacturer’s protocol with input of 100 ng DNA. The assay volume was always constant, and methylation levels were corrected for the mass of the DNA. Samples from the same individual but different treatments were run on the same plate.

### Library construction and whole-genome bisulphite sequencing (WGBS)

The construction of the library and bisulphite sequencing were conducted by BGI (Shenzhen, China). Briefly, DNA was fragmented by sonication using a Bioruptor (Diagenode, Belgium) to a mean size of *c.* 250 bp. This was followed by the blunt-ending, dA addition to the 3′-end, and finally, adaptor ligation, essentially according to the manufacturer’s instructions. Ligated DNA was subjected to bisulphite conversion using the EZ DNA Methylation-Gold kit (ZYMO). Following the administration of sodium bisulphite, unmethylated cytosine residues were converted to uracil, whereas 5-methylcytosine (5mC) remained unaffected. After the PCR amplification process, uracil residues were converted to thymine. Fragments of varying insert sizes were excised from a single lane of a 2% TAE agarose gel. The purification of the products was conducted using the QIAquick Gel Extraction Kit (Qiagen), while the amplification was achieved through PCR. Finally, sequencing was performed using the HighSeq4000 and 150-bp paired-end reads were generated.

The clean data were obtained after filtering and then mapped to the reference genome (assembly accession: GCF_018340385.1) for bioinformatics analysis by BSMAP. Subsequently, the duplication reads were removed, and the mapping results were merged according to each library. The mapping rate and bisulphite conversion rate were calculated for each sample. The methylation level was calculated by dividing the number of reads covering each mC by the total number of reads covering that cytosine [77], which was also equal to the mC/C ratio at each reference cytosine [78]. Putative differentially methylated regions (DMRs) were identified by comparing the control and treated sample methylomes using windows that contained at least five CpG (CHG or CHH) sites with a two-fold change in the methylation level and a Fisher test *P* value < 0.05. DMRs-related genes (DMGs) were annotated to pathways and processes using gene ontology (GO) and Kyoto Encyclopaedia of Genes and Genomes (KEGG) pathway analyses.

### Total RNA extraction and preparation

Total RNA was extracted from embryos using RNA isolation kits (Invitrogen, USA) in accordance with the instructions provided in the manual. The quality and integrity of the total RNA were ascertained using a Nanodrop spectrophotometer and an Agilent 2100 Bioanalyzer (Thermo Fisher Scientific, USA).

### RNA sequencing (RNA-Seq) and analysis

The mRNA was enriched using oligo (dT) magnetic beads and fragmented to synthesise cDNA for PCR amplification. Then, the PCR products were denatured into single strand to develop the single-stranded circular DNA library and sequenced using DNBSEQ (BGI, China). Clean reads were obtained after filtering the raw data and mapped to the reference genome (GCF_018340385.1) for further bioinformatics analysis. Clean reads mapping to the reference genome was conducted using HISAT2 software [79] and followed by StringTie [80]. The assembled results of all samples were combined with Cuffmerge. Bowtie2 software was used to align clean reads to the reference sequence (Langmead and Salzberg, 2012). The differentially expressed genes (DEGs) were identified using the DEseq2 method [81] with the fold change ≥ 2, and the corrected *P* value (*Q* value) ≤ 0.001. GO and KEGG pathway enrichment analyses were performed by the Phyper function in R Project to obtain the annotation of these genes for analysing gene function. The *Q* value ≤ 0.05 was considered significant.

### Quantitative proteomics in embryos and bioinformatic analyses

Thirty embryos from each group were pooled and homogenised by a grinder with steel beads, containing 1X Cocktail, EDTA and SDS L3. The supernatant was extracted following centrifugation (25,000 g, 4 °C for 15 min) and reduced with 10 mM DTT for 30 min at 37 °C. Samples were alkylated with iodoacetamide (55 mM final concentration) in the dark for 45 min. The treated samples were precipitated in cold acetone at a ratio of 1:5 for 2 h at − 20 °C. After centrifugation at 25,000 g, the dry pellet was dissolved in 0.5 M triethylammonium bicarbonate (TEAB) (Applied Biosystems). Bradford assay and SDS-PAGE were used for quality control of the protein extraction. For protein enzymatic hydrolysis, 100 μg of protein solution per sample was diluted with 50 mM NH_4_HCO_3_ and digested with trypsin enzyme. Enzymatic peptides were desalted using a Strata X column and vacuumed to dryness. The drained peptide samples were reconstituted with mobile phase A, and the supernatant was injected for separation using a Thermo EASY-nLC1200 system. The sample was enriched, desalted and separated on a C18 column with a gradient of mobile phase B at a flow rate of 200 nL/min, then directly connected to a mass spectrometer for detection. The peptides separated by liquid chromatography were ionised using a nanoESI source and detected in the DIA mode on an Orbitrap Eclipse mass spectrometer. Key settings included a 2.0 kV ion source voltage, 400–1250 m/z scan range, 120,000 resolution, HCD fragmentation at 30% energy and a maximum ion implantation time of 90 ms.

The MS/MS spectra were queried against the protein database (UniProt protein database and protein databases based on genome annotation from NCBI and Ensembl) of common carp with MASCOT software (Matrix Science, London, UK). Software Scaffold (Proteome Software Inc., Portland, OR, USA) was used to quantify and analyse the data derived from the MS/MS intensities of the reporter tags. A 1.5-fold change was set as the threshold, and *P* < 0.05 was used to identify significantly differentially expressed proteins (DEPs). Gene ontology classification was studied using PANTHER (http://pantherdb.org/), and pathway enrichment was conducted by Partek Genomics Suite 6.6 (Partek, Inc., St Louis, MO, USA).

### Statistical analysis

The normality of the distribution and homogeneity of variances for all data were evaluated using Shapiro–Wilk and Levene’s tests. Statistical significance of sperm phenotypic parameters, mdC/dC (%), was tested using either one-way ANOVA followed by a Tukey test or the nonparametric Kruskal–Wallis test followed by a Dunn pairwise comparison. All statistical analyses were conducted using GraphPad Prism 9.1.0 (GraphPad Software, San Diego, CA, USA) and the R programming language. Results are expressed as means ± SD. Differences were considered significant at *P* < 0.05 except for special instructions.

## Supplementary Information

Additional file 1: Fig. S1 Effects of short-term storage on sperm straight-line velocity (VSL; A), average path velocity (VAP; B), path linearity (LIN = VSL/VCL; C), wobble (WOB; D), progressive motility (PROG; E) and flagellar beating frequency (BCF, F) in common carp (*Cyprinus carpio*). Fig. S2 Effects of sperm storage on global methylation patterns and differentially methylated regions (DMRs) in both sperm and embryos derived from this sperm. Fig. S3 Kyoto Encyclopaedia of Genes and Genomes (KEGG) enrichment scatter plot for the differential expression genes (DEG)-related pathway. Fig. S4 Effects of short-term sperm storage on differentially expressed genes (DEGs) and RNA expression in the resulting mid-blastula embryos.

Additional file 2: WGBS quality and differentially methylated genes in sperm and offspring.

Additional file 3: Gene ontology analysis of DMRs. File containing GO functional enrichment analysis of observed DMRs between fresh and aged sperm groups.

Additional file 4: Gene ontology analysis of DMRs. File containing GO functional enrichment analysis of observed DMRs between offsprings derived from fresh and aged sperm groups.

Additional file 5: Summary of RNA-Seq quality assessment, differentially expressed genes, GO enrichment results and KEGG pathway analysis.

Additional file 6: Protein identification, differential expression and GO enrichment in larvae and MBT-stage embryos.

Additional file 7: Common DMR-associated genes and transcriptomic overlap in F0 sperm and F1 offspring.

## Data Availability

The raw data files and the processed methylation data are publicly available at the Gene Expression Omnibus (GEO) under accession number GSE283590 and GSE283591 [82, 83]. Additional data and supplementary figures are included in the supplementary files of this publication. All data generated or analysed during this study are included in this published article and its supplementary information files.

## Abbreviations

WGBS: Whole-genome bisulphite sequencing
DPS: Days post-stripping
PBS: Phosphate buffered saline
DMRs: Differentially methylated regions
DMGs: Differentially methylated genes
DEGs: Differentially expressed genes
dph: Days post-hatching
dpf: Days post-fertilisation
hpf: Hours post-fertilisation
ART: Assisted reproductive technology
RNA-Seq: RNA sequencing
PCA: Principal component analysis
MOT: Percentage of motile sperm
VSL: Straight-line velocity
VCL: Curvilinear velocity
VAP: Average path velocity
LIN: Linearity
WOB: Wobble
PROG: Progression
BCF: Beat cross frequency
PS: Physiological solution
MBT: Mid-blastula transition
GO: Gene ontology
KEGG: Kyoto Encyclopaedia of Genes and Genomes
TSS: Transcriptional start sites
TES: Transcription end sites
DEPs: Differentially expressed proteins
PPI: Protein-protein interaction
uba2: Ubiquitin-like modifier-activating enzyme 2
ncln: Nicalin
vtg: Vitellogenin

## References

[1] Wyck S, Herrera C, Requena CE, Bittner L, Hajkova P, Bollwein H, et al. Oxidative stress in sperm affects the epigenetic reprogramming in early embryonic development. Epigenetics Chromatin. 2018;11(1): 60. 30333056 10.1186/s13072-018-0224-y PMC6192351

[2] Brionne A, Goupil AS, Kica S, Lareyre JJ, Labbé C, Laurent A. Spermatozoa methylome and its sensitivity to water temperature in a teleost fish. Sci Total Environ. 2023;892: 164077. 37257597 10.1016/j.scitotenv.2023.164077

[3] Liu A, Zeng F, Wang L, Zhen H, Xia X, Pei H, et al. High temperature influences *DNA* methylation and transcriptional profiles in sea urchins (*Strongylocentrotus intermedius*). BMC Genomics. 2023;24(1): 491. 37641027 10.1186/s12864-023-09616-7 PMC10464075

[4] Khezri A, Narud B, Stenseth EB, Johannisson A, Myromslien FD, Gaustad AH, et al. DNA methylation patterns vary in boar sperm cells with different levels of DNA fragmentation. BMC Genomics. 2019;20(1): 897. 31775629 10.1186/s12864-019-6307-8 PMC6880426

[5] Song B, Wang C, Chen Y, Li G, Gao Y, Zhu F, et al. Sperm DNA integrity status is associated with DNA methylation signatures of imprinted genes and non-imprinted genes. J Assist Reprod Genet. 2021;38(8):2041–8. 33786731 10.1007/s10815-021-02157-6 PMC8417181

[6] Skvortsova K, Iovino N, Bogdanović O. Functions and mechanisms of epigenetic inheritance in animals. Nat Rev Mol Cell Biol. 2018;19:774–90. 30425324 10.1038/s41580-018-0074-2

[7] Perez MF, Lehner B. Intergenerational and transgenerational epigenetic inheritance in animals. Nat Cell Biol. 2019;21(2):143–51. 30602724 10.1038/s41556-018-0242-9

[8] Iwanami N, Lawir DF, Sikora K, O´Meara C, Takeshita K, Schorpp M, et al. Transgenerational inheritance of impaired larval T cell development in zebrafish. Nat Commun. 2020;11(1): 4505. 32908148 10.1038/s41467-020-18289-9 PMC7481223

[9] Angers B, Castonguay E, Massicotte R. Environmentally induced phenotypes and DNA methylation: how to deal with unpredictable conditions until the next generation and after. Mol Ecol. 2010;19(7):1283–95. 20298470 10.1111/j.1365-294X.2010.04580.x

[10] Richard Albert J, Au Yeung WK, Toriyama K, Kobayashi H, Hirasawa R, Brind’Amour J, et al. Maternal DNMT3A-dependent de novo methylation of the paternal genome inhibits gene expression in the early embryo. Nat Commun. 2020;11(1):5417. 33110091 10.1038/s41467-020-19279-7 PMC7591512

[11] Tai Z, Guan P, Zhang T, Liu W, Li L, Wu Y, et al. Effects of parental environmental copper stress on offspring development: DNA methylation modification and responses of differentially methylated region-related genes in transcriptional expression. J Hazard Mater. 2022;424: 127600. 34801305 10.1016/j.jhazmat.2021.127600

[12] Jeremias G, Veloso T, Gonçalves FJM, Van Nieuwerburgh F, Pereira JL, Asselman J. Multigenerational DNA methylation responses to copper exposure in *Daphnia*: potential targets for epigenetic biomarkers? Chemosphere. 2022;308: 136231. 36055596 10.1016/j.chemosphere.2022.136231

[13] Lismer A, Kimmins S. Emerging evidence that the mammalian sperm epigenome serves as a template for embryo development. Nat Commun. 2023;14(1): 2142. 37059740 10.1038/s41467-023-37820-2 PMC10104880

[14] Cheng Y, Vechtova P, Fussy Z, Sterba J, Linhartová Z, Rodina M, et al. Changes in phenotypes and DNA methylation of *in vitro* aging sperm in common carp *Cyprinus carpio*. Int J Mol Sci. 2021;22(11): 5925. 34073009 10.3390/ijms22115925 PMC8198300

[15] Cheng Y, Zhang S, Nayak R, Věchtová P, Schumacher F, Linhartová P, et al. Fertilization by short-term stored sperm alters DNA methylation patterns at single-base resolution in common carp (*Cyprinus carpio*) embryos. Rev Fish Biol Fish. 2024;34(3):1167–87.

[16] Heard E, Martienssen RA. Transgenerational epigenetic inheritance: myths and mechanisms. Cell. 2014;157(1):95–109. 24679529 10.1016/j.cell.2014.02.045 PMC4020004

[17] Zhang S, Cheng Y, Věchtová P, Boryshpolets S, Shazada NE, Alavi SMH, et al. Potential implications of sperm DNA methylation functional properties in aquaculture management. Rev Aquacult. 2023;15(2):536–56.

[18] Jones PA. Functions of DNA methylation: islands, start sites, gene bodies and beyond. Nat Rev Genet. 2012;13(7):484–92. 22641018 10.1038/nrg3230

[19] Zhang H, Lang Z, Zhu JK. Dynamics and function of DNA methylation in plants. Nat Rev Mol Cell Biol. 2018;19(8):489–506. 29784956 10.1038/s41580-018-0016-z

[20] He L, Liang X, Wang Q, Yang C, Li Y, Liao L, et al. Genome-wide DNA methylation reveals potential epigenetic mechanism of age-dependent viral susceptibility in grass carp. Immun Ageing. 2022;19(1): 28. 35655223 10.1186/s12979-022-00285-w PMC9161582

[21] Smith ZD, Chan MM, Mikkelsen TS, Gu H, Gnirke A, Regev A, et al. A unique regulatory phase of DNA methylation in the early mammalian embryo. Nature. 2012;484(7394):339–44. 22456710 10.1038/nature10960 PMC3331945

[22] Jiang L, Zhang J, Wang JJ, Wang L, Zhang L, Li G, et al. Sperm, but not oocyte, DNA methylome is inherited by zebrafish early embryos. Cell. 2013;153(4):773–84. 23663777 10.1016/j.cell.2013.04.041 PMC4081501

[23] Potok ME, Nix DA, Parnell TJ, Cairns BR. Reprogramming the maternal zebrafish genome after fertilization to match the paternal methylation pattern. Cell. 2013;153(4):759–72. 23663776 10.1016/j.cell.2013.04.030 PMC4030421

[24] Radford EJ, Ito M, Shi H, Corish JA, Yamazawa K, Isganaitis E, et al. In utero undernourishment perturbs the adult sperm methylome and intergenerational metabolism. Science. 2014;345(6198): 1255903. 25011554 10.1126/science.1255903 PMC4404520

[25] Anway MD, Cupp AS, Uzumcu N, Skinner MK. Toxicology: epigenetic transgenerational actions of endocrine disruptors and male fertility. Science (80-). 2005;308(5727):1466–9. 10.1126/science.1108190 PMC11423801 15933200

[26] Newbold RR, Padilla-Banks E, Jefferson WN. Adverse effects of the model environmental estrogen diethylstilbestrol are transmitted to subsequent generations. Endocrinology. 2006;147(6):S11-7. 16690809 10.1210/en.2005-1164

[27] Wang SY, Lau K, Lai KP, Zhang JW, Tse ACK, Li JW, et al. Hypoxia causes transgenerational impairments in reproduction of fish. Nat Commun. 2016;7: 12114. 27373813 10.1038/ncomms12114 PMC4932196

[28] Di Santo M, Tarozzi N, Nadalini M, Borini A. Human sperm cryopreservation: update on techniques, effect on DNA integrity, and implications for ART. Adv Urol. 2012. 10.1155/2012/854837. 22194740 10.1155/2012/854837 PMC3238352

[29] Magnotti C, Cerqueira V, Lee-Estevez M, Farias JG, Valdebenito I, Figueroa E. Cryopreservation and vitrification of fish semen: a review with special emphasis on marine species. Rev Aquacult. 2018;10(1):15–25.

[30] Contreras P, Dumorné K, Ulloa-Rodríguez P, Merino O, Figueroa E, Farías JG, et al. Effects of short-term storage on sperm function in fish semen: a review. Rev Aquacult. 2020;12(3):1373–89.

[31] Shazada NE, Alavi SMH, Siddique MAM, Cheng Y, Zhang S, Rodina M, et al. Short-term storage of sperm in common carp from laboratory research to commercial production—a review. Rev Aquacult. 2024;16(1):174–89.

[32] Gasparini C, Dosselli R, Evans JP. Sperm storage by males causes changes in sperm phenotype and influences the reproductive fitness of males and their sons. Evol Lett. 2017;1(1):16–25. 30283635 10.1002/evl3.2 PMC6121797

[33] Vallet-Buisan M, Mecca R, Jones C, Coward K, Yeste M. Contribution of semen to early embryo development: fertilization and beyond. Hum Reprod Update. 2023;29(4):395–433. 36882116 10.1093/humupd/dmad006

[34] Dahlet T, Argüeso Lleida A, Al Adhami H, Dumas M, Bender A, Ngondo RP, et al. Genome-wide analysis in the mouse embryo reveals the importance of DNA methylation for transcription integrity. Nat Commun. 2020;11(1): 3153. 32561758 10.1038/s41467-020-16919-w PMC7305168

[35] Depincé A, Gabory A, Dziewulska K, Le Bail PY, Jammes H, Labbé C. DNA methylation stability in fish spermatozoa upon external constraint: impact of fish hormonal stimulation and sperm cryopreservation. Mol Reprod Dev. 2020;87(1):124–34. 31746511 10.1002/mrd.23297

[36] Waghmare SG, Samarin AM, Samarin AM, Danielsen M, Møller HS, Policar T, et al. Histone acetylation dynamics during in vivo and in vitro oocyte aging in common carp *Cyprinus carpio*. Int J Mol Sci. 2021;22(11): 6036. 34204879 10.3390/ijms22116036 PMC8199789

[37] Cheng Y, Waghmare SG, Zhang S, Vechtová P, Schumacher F, Kleuser B, et al. Aging of common carp (*Cyprinus carpio* L.) sperm induced by short-term storage does not alter global DNA methylation and specific histone modifications in offspring. Aquaculture. 2023;571: 739484.

[38] El Kamouh M, Brionne A, Sayyari A, Laurent A, Labbé C. Cryopreservation effect on DNA methylation profile in rainbow trout spermatozoa. Sci Rep. 2023;13(1): 19029. 37923780 10.1038/s41598-023-44803-2 PMC10624875

[39] Cejko BI, Horváth Á, Kollár T, Kása E, Lujić J, Marinović Z, et al. Optimisation of sodium and potassium concentrations and pH in the artificial seminal plasma of common carp *Cyprinus carpio* L. Fish Physiol Biochem. 2018;44(6):1435–42. 29560576 10.1007/s10695-018-0491-3 PMC6294815

[40] Cejko BI, Żarski D, Sarosiek B, Dryl K, Palińska-Żarska K, Skorupa W, et al. Application of artificial seminal plasma to short-term storage of a large volume of common carp (*Cyprinus carpio*) sperm for two weeks under controlled conditions. Aquaculture. 2022;546: 737385.

[41] Cheng Y, Zhang S, Linhartová Z, Shazada NE, Linhart O. Common carp (*Cyprinus carpio*) sperm reduction during short-term *in vitro* storage at 4 °C. Anim Reprod Sci. 2022;243: 107017. 35714400 10.1016/j.anireprosci.2022.107017

[42] Zhang S, Cheng Y, Alavi SMH, Shazada NE, Linhartová Z, Rodinová V, et al. Elevated temperature promotes spermatozoa motility kinetics and fertilizing ability following short-term storage: an implication for artificial reproduction of common carp *Cyprinus carpio* in a hatchery. Aquaculture. 2023;565: 739126.

[43] Neuhauss SCF. Zebrafish vision: structure and function of the zebrafish visual system. Fish Physiol. 2010;29:81–122.

[44] Nelson JC, Granato M. Zebrafish behavior as a gateway to nervous system assembly and plasticity. Development. 2022;149(9): dev177998. 35552393 10.1242/dev.177998 PMC9148562

[45] Pérez-Cerezales S, Martínez-Páramo S, Beirão J, Herráez MP. Fertilization capacity with rainbow trout DNA-damaged sperm and embryo developmental success. Reproduction. 2010;139(6):989–97. 20357047 10.1530/REP-10-0037

[46] Donnelly ET, McClure N, Lewis SEM. Cryopreservation of human semen and prepared sperm: effects on motility parameters and DNA integrity. Fertil Steril. 2001;76(5):892–900. 11704107 10.1016/s0015-0282(01)02834-5

[47] Riel JM, Yamauchi Y, Huang TTF, Grove J, Ward MA. Short-term storage of human spermatozoa in electrolyte-free medium without freezing maintains sperm chromatin integrity better than cryopreservation. Biol Reprod. 2011;85(3):536–47. 21593474 10.1095/biolreprod.111.091322 PMC3159537

[48] Sanchez-Partida LG, Maginnis G, Dominko T, Martinovich C, McVay B, Fanton J, et al. Live rhesus offspring by artificial insemination using fresh sperm and cryopreserved sperm. Biol Reprod. 2000;63(4):1092–7. 10993831 10.1095/biolreprod63.4.1092

[49] Boe-Hansen GB, Ersbøll AK, Greve T, Christensen P. Increasing storage time of extended boar semen reduces sperm DNA integrity. Theriogenology. 2005;63(7):2006–19. 15823356 10.1016/j.theriogenology.2004.09.006

[50] Sharma R, Kattoor AJ, Ghulmiyyah J, Agarwal A. Effect of sperm storage and selection techniques on sperm parameters. Syst Biol Reprod Med. 2015;61(1):1–12. 25354153 10.3109/19396368.2014.976720

[51] Dietrich MA, Judycka S, Słowińska M, Kodzik N, Ciereszko A. Short-term storage-induced changes in the proteome of carp (*Cyprinus carpio* L.) spermatozoa. Aquaculture. 2021;530: 735784.

[52] Dzyuba V, Giebułtowicz J, Dzyuba B, Fedorova G, Kholodnyy V, Kowalski RK, et al. Changes in sperm metabolome during carp sperm short-term storage in different media: in search of sperm quality and storage capability markers. Aquaculture. 2024;581: 740381.

[53] Panda A, Judycka S, Palińska-Żarska K, Debernardis R, Jarmołowicz S, Jastrzębski JP, et al. Paternal-effect-genes revealed through sperm cryopreservation in *Perca fluviatilis*. Sci Rep. 2024;14(1): 6396. 38493223 10.1038/s41598-024-56971-w PMC10944473

[54] Fernández-Díez C, Herráez MP. Changes in transcriptomic profile of trout larvae obtained with frozen sperm. Aquaculture. 2018;492:306–20.

[55] Rønnestad I, Yúfera M, Ueberschär B, Ribeiro L, Sæle Ø, Boglione C. Feeding behaviour and digestive physiology in larval fish: current knowledge, and gaps and bottlenecks in research. Rev Aquac. 2013;5:S59–98.

[56] Nusbaumer D, Da Cunha LM, Wedekind C. Sperm cryopreservation reduces offspring growth. Proc R Soc B Biol Sci. 1911;2019(286):20191644. 10.1098/rspb.2019.1644 PMC6784727 31551057

[57] Tse WKF, You MS, Ho SHK, Jiang YJ. The deubiquitylating enzyme, Cops6, regulates different developmental processes during early zebrafish embryogenesis. Int J Dev Biol. 2011;55(1):19–24. 21425078 10.1387/ijdb.103089wt

[58] Lewno MT, Cui T, Wang X. Cullin deneddylation suppresses the necroptotic pathway in cardiomyocytes. Front Physiol. 2021;12: 690423. 34262479 10.3389/fphys.2021.690423 PMC8273387

[59] Shin DG, Bayarsaihan D. A novel epi-drug therapy based on the suppression of BET family epigenetic readers. Yale J Biol Med. 2017;90(1):63–71. 28356894 PMC5369046

[60] Arking DE, Junttila MJ, Goyette P, Huertas-Vazquez A, Eijgelsheim M, Blom MT, et al. Identification of a sudden cardiac death susceptibility locus at 2q24.2 through genome-wide association in European ancestry individuals. PLoS Genet. 2011;7(6): e1002158. 21738491 10.1371/journal.pgen.1002158 PMC3128111

[61] Corsten-Janssen N, Scambler PJ. Clinical and molecular effects of CHD7 in the heart. Am J Med Genet C Semin Med Genet. 2017;175(4):487–95. 29088513 10.1002/ajmg.c.31590

[62] Fischer A, Schumacher N, Maier M, Sendtner M, Gessler M. The notch target genes Hey1 and Hey2 are required for embryonic vascular development. Genes Dev. 2004;18(8):901–11. 15107403 10.1101/gad.291004 PMC395849

[63] Nemir M, Croquelois A, Pedrazzini T, Radtke F. Induction of cardiogenesis in embryonic stem cells via downregulation of Notch1 signaling. Circ Res. 2006;98(12):1471–8. 16690879 10.1161/01.RES.0000226497.52052.2a

[64] Levkowitz G, Zeller J, Sirotkin HI, French D, Schilbach S, Hashimoto H, et al. Zinc finger protein too few controls the development of monoaminergic neurons. Nat Neurosci. 2003;6(1):28–33. 12469125 10.1038/nn979

[65] Dawson MS, Gordon-Fleet K, Yan L, Tardos V, He H, Mui K, et al. Sexual dimorphism in the social behaviour of *Cntnap2*-null mice correlates with disrupted synaptic connectivity and increased microglial activity in the anterior cingulate cortex. Commun Biol. 2023;6(1): 846. 37582968 10.1038/s42003-023-05215-0 PMC10427688

[66] Kosteria I, Tsangaris GT, Gkourogianni A, Anagnostopoulos A, Papadopoulou A, Papassotiriou I, et al. Proteomics of children born after intracytoplasmic sperm injection reveal indices of an adverse cardiometabolic profile. J Endocr Soc. 2017;1(4):288–301. 29264487 10.1210/js.2016-1052 PMC5686695

[67] La Rovere M, Franzago M, Stuppia L. Epigenetics and neurological disorders in ART. Int J Mol Sci. 2019;20(17): 4169. 31454921 10.3390/ijms20174169 PMC6747212

[68] Lismer A, Dumeaux V, Lafleur C, Lambrot R, Brind’Amour J, Lorincz MC, et al. Histone H3 lysine 4 trimethylation in sperm is transmitted to the embryo and associated with diet-induced phenotypes in the offspring. Dev Cell. 2021;56(5):671-686.e6. 33596408 10.1016/j.devcel.2021.01.014

[69] Linhart O, Rodina M, Kašpar V. Common carp (*Cyprinus carpio* Linneaus, 1758) male fertilization potency with secure number of spermatozoa per ova. J Appl Ichthyol. 2015;31(S1):169–73.

[70] Wilson-Leedy JG, Ingermann RL. Development of a novel CASA system based on open source software for characterization of zebrafish sperm motility parameters. Theriogenology. 2007;67(3):661–72. 17137620 10.1016/j.theriogenology.2006.10.003

[71] Flajšhans M, Cosson J, Rodina M, Linhart O. The application of image cytometry to viability assessment in dual fluorescence-stained fish spermatozoa. Cell Biol Int. 2004;28(12):955–9. 15566965 10.1016/j.cellbi.2004.07.014

[72] Li P, Dzyuba B, Hulak M, Rodina M, Boryshpolets S, Li ZH, et al. Percoll gradient separation of cryopreserved common carp spermatozoa to obtain a fraction with higher motility, velocity and membrane integrity. Theriogenology. 2010;74(8):1356–61. 20688378 10.1016/j.theriogenology.2010.06.005

[73] Perchec G, Cosson MP, Cosson J, Jeulin C, Billard R. Morphological and kinetic changes of carp (*Cyprinus carpio*) spermatozoa after initiation of motility in distilled water. Cell Motil Cytoskeleton. 1996;35(2):113–20. 8894281 10.1002/(SICI)1097-0169(1996)35:2<113::AID-CM4>3.0.CO;2-B

[74] Cheng Y, Xin M, Gela D, Rodina M, Tučková V, Kašpar V, et al. Optimization of sterlet (*Acipenser ruthenus*) egg incubation. Anim Reprod Sci. 2020;215: 106334. 32216936 10.1016/j.anireprosci.2020.106334

[75] Schneider CA, Rasband WS, Eliceiri KW. NIH image to ImageJ: 25 years of image analysis. Nat Methods. 2012;9(7):671–5. 22930834 10.1038/nmeth.2089 PMC5554542

[76] Natarajan VP, Zhang X, Morono Y, Inagaki F, Wang F. A modified SDS-based DNA extraction method for high quality environmental DNA from seafloor environments. Front Microbiol. 2016;7: 986. 27446026 10.3389/fmicb.2016.00986 PMC4917542

[77] Xiang H, Zhu J, Chen Q, Dai F, Li X, Li M, et al. Single base-resolution methylome of the silkworm reveals a sparse epigenomic map. Nat Biotechnol. 2010;28(5):516–20. 20436463 10.1038/nbt.1626

[78] Lister R, Pelizzola M, Dowen RH, Hawkins RD, Hon G, Tonti-Filippini J, et al. Human DNA methylomes at base resolution show widespread epigenomic differences. Nature. 2009;462(7271):315–22. 19829295 10.1038/nature08514 PMC2857523

[79] Kim D, Langmead B, Salzberg SL. HISAT: a fast spliced aligner with low memory requirements. Nat Methods. 2015;12(4):357–60. 25751142 10.1038/nmeth.3317 PMC4655817

[80] Pertea M, Pertea GM, Antonescu CM, Chang TC, Mendell JT, Salzberg SL. Stringtie enables improved reconstruction of a transcriptome from RNA-seq reads. Nat Biotechnol. 2015;33(3):290–5. 25690850 10.1038/nbt.3122 PMC4643835

[81] Wang L, Feng Z, Wang X, Wang X, Zhang X. DEGseq: an R package for identifying differentially expressed genes from RNA-seq data. Bioinformatics. 2009;26(1):136–8. 19855105 10.1093/bioinformatics/btp612

[82] Cheng Y, Alavi SMH, Zhang S, Nayak R, Waghmare SG, Rahi RD, Shazada N, Ma Z, Linhart O, Linhartová Z. Comprehensive multi-omics analysis uncovers potential risks of aged sperm on offspring development after short-term storage. NCBI GEO; 2025a. https://www.ncbi.nlm.nih.gov/geo/query/acc.cgi?acc=GSE283590. Accessed 8 Jul 2025. 10.1186/s12915-025-02379-5 PMC12374335 40846931

[83] Cheng Y, Alavi SMH, Zhang S, Nayak R, Waghmare SG, Rahi RD, Shazada N, Ma Z, Linhart O, Linhartová Z. Comprehensive multi-omics analysis uncovers potential risks of aged sperm on offspring development after short-term storage. NCBI GEO; 2025b. https://www.ncbi.nlm.nih.gov/geo/query/acc.cgi?acc=GSE283591. Accessed 8 Jul 2025. 10.1186/s12915-025-02379-5 PMC12374335 40846931

