# Supplementary material for: Comprehensive multi-omics analysis uncovers potential risks of aged sperm on offspring development after short-term storage
Source: BMC Biol. 2025 Aug 22;23:264. doi: 10.1186/s12915-025-02379-5 (PMC12374335; doi:10.1186/s12915-025-02379-5)
Supplement: Supplementary file 1 — Additional file 1: Fig. S1 Effects of short-term storage on sperm straight-line velocity (VSL; A), average path velocity (VAP; B), path linearity (LIN = VSL/VCL; C), wobble (WOB; D), progressive motility (PROG; E) and flagellar beating frequency (BCF, F) in common carp (Cyprinus carpio). Fig. S2 Effects of sperm storage on global methylation patterns and differentially methylated regions (DMRs) in both sperm and embryos derived from this sperm. Fig. S3 Kyoto Encyclopaedia of Genes and Genomes (KEGG) enrichment scatter plot for the differential expression genes (DEG)-related pathway. Fig. S4 Effects of short-term sperm storage on differentially expressed genes (DEGs) and RNA expression in the resulting mid-blastula embryos. [file 12915_2025_2379_MOESM1_ESM.docx]

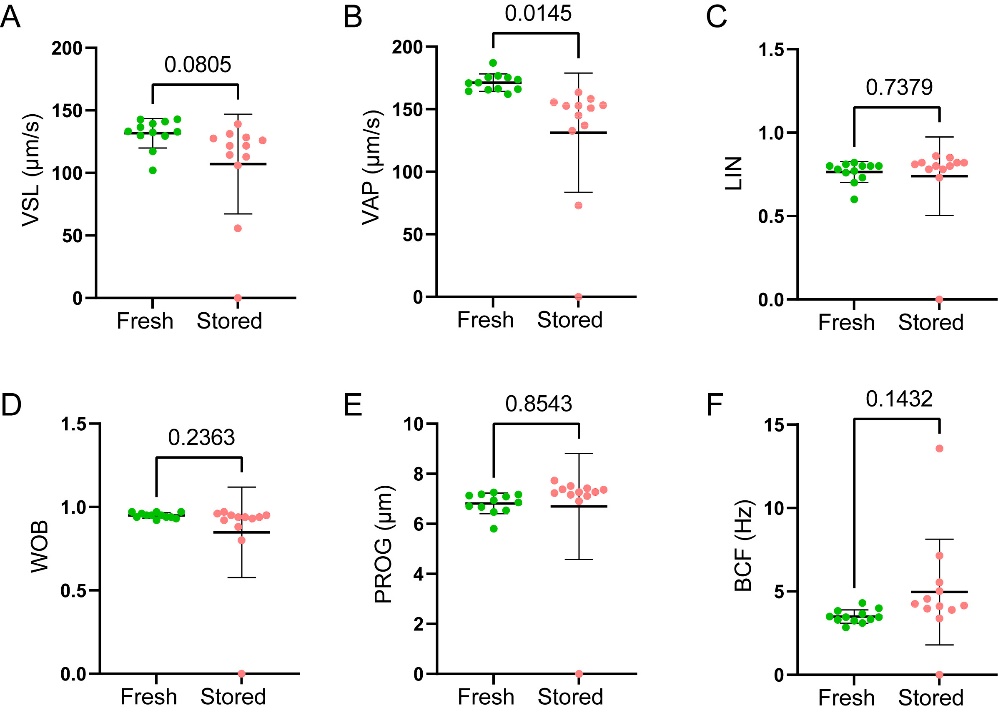


**Fig. S1.** Effects of short-term storage on sperm straight-line velocity (VSL; **A**), average path velocity (VAP; **B**), path linearity (LIN = VSL / VCL; **C**), wobble (WOB; **D**), progressive motility (PROG; **E**) and flagellar beating frequency (BCF, **F**) in common carp (*Cyprinus carpio*) (***n* = 12**). Sperm was stored on ice for 14 days, and motility analysis was performed using a CASA. Results are expressed as means ± S.D. with actual *P*-values showing significant differences at *P* < 0.05.


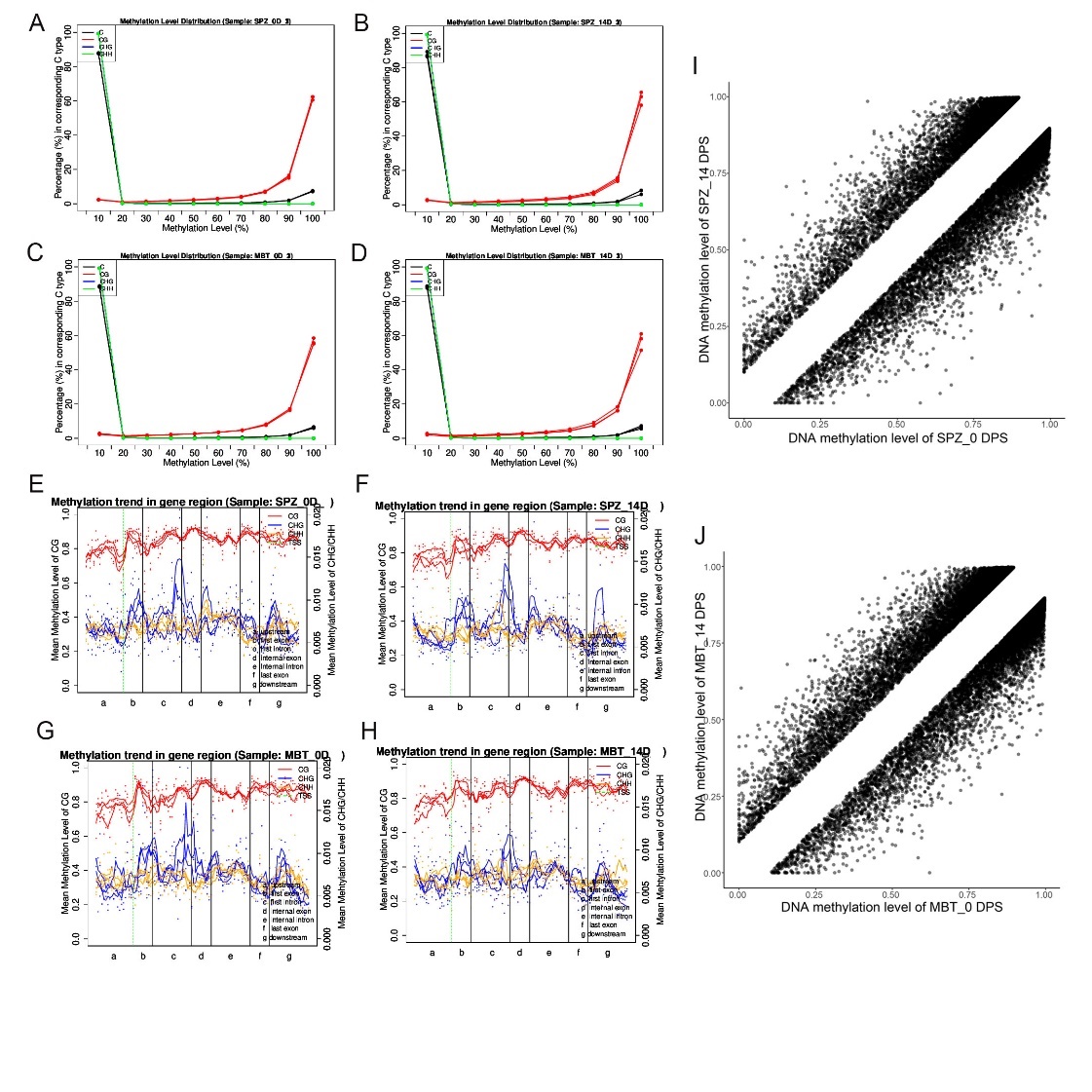


**Fig. S2.** Effects of sperm storage on global methylation patterns and differentially methylated regions (DMRs) in both sperm and embryos derived from this sperm. (**A)** and **(B)** show distribution of genome wide CpG methylation levels in fresh and stored sperm, respectively. (**C)** and (**D)** show distribution of genome wide CpG methylation levels in the embryos from fresh and stored sperm, respectively. (**E**) and (**F)** show the methylation levels in the different gene regions in fresh and stored sperm, respectively. (**G)** and **(H)** show the methylation levels in the different gene regions in the embryos resulting from fresh and stored sperm, respectively. (**I)** and (**J)** show scatter plots for hyper- and hypoDMRs in sperm and in their resulting embryos, respectively.


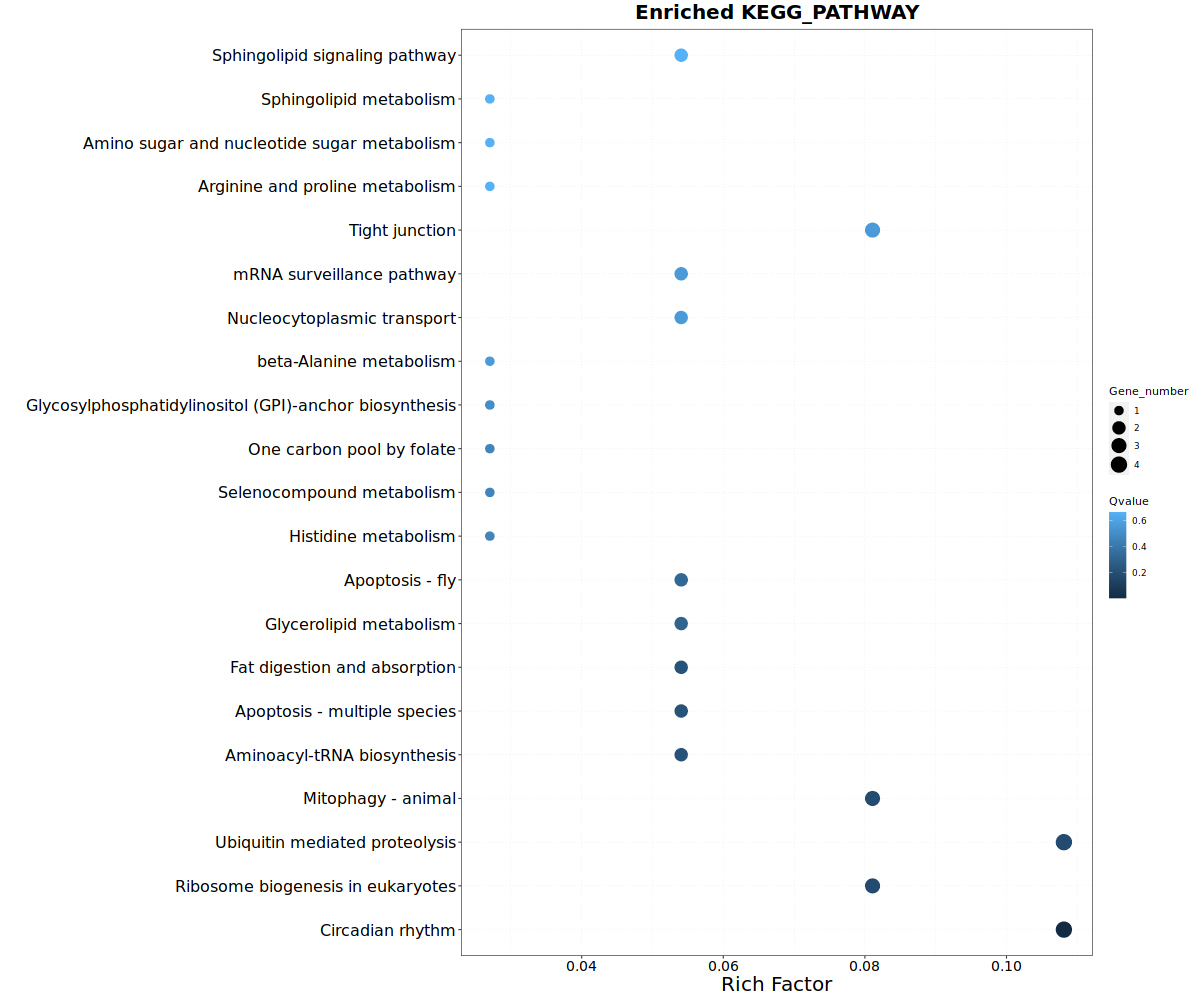


**Fig. S3.** Kyoto Encyclopedia of Genes and Genomes (KEGG) enrichment scatter plot for the differential expression genes (DEG)-related pathway. The *x*-axis represents the rich factor, and the *y*-axis represents the pathway name. The size of points stands for DEG-related gene counts and the colour intensity represents a different *q*-value.


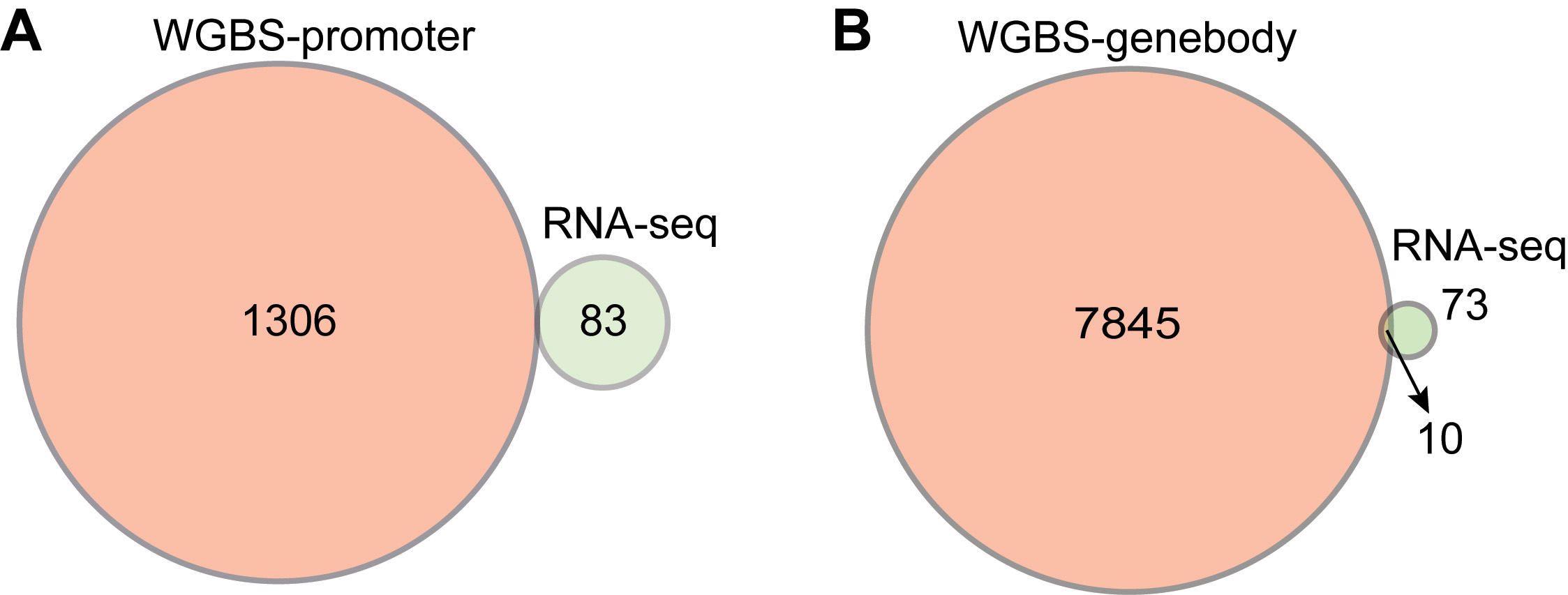


**Fig. S4.** Effects of short-term sperm storage on differentially expressed genes (DEGs) and RNA expression in the resulting mid-blastula embryos. Venn diagrams show common genes between differentially methylated regions (DMRs) identified by whole-genome bisulfite sequencing (WGBS) in (**A**) promoter regions and (**B**) gene bodies; DEGs identified by RNA sequencing (RNA-Seq).
